# A Dynamic Neural Field Model of Mesoscopic Cortical Activity Captured with Voltage-Sensitive Dye Imaging

**DOI:** 10.1371/journal.pcbi.1000919

**Published:** 2010-09-09

**Authors:** Valentin Markounikau, Christian Igel, Amiram Grinvald, Dirk Jancke

**Affiliations:** 1 Institut für Neuroinformatik, Ruhr-Universität Bochum, Bochum, Germany; 2 The International Graduate School of Neuroscience, Ruhr-Universität Bochum, Bochum, Germany; 3 Department of Neurobiology, Weizmann Institute of Science, Rehovot, Israel; 4 Cognitive Neurobiology, Ruhr-Universität Bochum, Bochum, Germany; Indiana University, United States of America

## Abstract

A neural field model is presented that captures the essential non-linear characteristics of activity dynamics across several millimeters of visual cortex in response to local flashed and moving stimuli. We account for physiological data obtained by voltage-sensitive dye (VSD) imaging which reports mesoscopic population activity at high spatio-temporal resolution. Stimulation included a single flashed square, a single flashed bar, the line-motion paradigm – for which psychophysical studies showed that flashing a square briefly before a bar produces sensation of illusory motion within the bar – and moving squares controls. We consider a two-layer neural field (NF) model describing an excitatory and an inhibitory layer of neurons as a coupled system of non-linear integro-differential equations. Under the assumption that the aggregated activity of both layers is reflected by VSD imaging, our phenomenological model quantitatively accounts for the observed spatio-temporal activity patterns. Moreover, the model generalizes to novel similar stimuli as it matches activity evoked by moving squares of different speeds. Our results indicate that feedback from higher brain areas is not required to produce motion patterns in the case of the illusory line-motion paradigm. Physiological interpretation of the model suggests that a considerable fraction of the VSD signal may be due to inhibitory activity, supporting the notion that balanced intra-layer cortical interactions between inhibitory and excitatory populations play a major role in shaping dynamic stimulus representations in the early visual cortex.

## Author Summary

Understanding the functioning of the primary visual cortex requires characterization of the non-linear dynamics that underlie visual perception and of how the cortical architecture gives rise to these dynamics. Recent advances in real-time voltage-sensitive dye (VSD) imaging permit recording of cortical population activity with high spatial and temporal resolution. This wealth of data can be related to cortical function, dynamics, and architecture by computational modeling. Here we used a mesoscopic neural field model to describe brain dynamics at the population level as measured by VSD imaging. Introduced in 1972 by Wilson and Cowan, these models are derived from statistical mechanics to analyze the collective properties of large numbers of neurons. For simplicity, the cortical planar tissue is assumed to contain only two types of homogeneously distributed neurons (excitatory and inhibitory) that interact via recurrent lateral connections. This study shows 1) how a concise neural field model can simulate VSD data quantitatively in space and time by identifying the underlying non-linear dynamics, 2) how such a model can support hypotheses about visual information processing, and 3) how the model can be linked to the neuronal architecture.

## Introduction

Visual cortical activity does not exclusively mirror visual input but rather reflects the contribution of additional recurrent processes involving lateral and local feedback couplings. Understanding cortical processing requires a theoretical understanding of the underlying activity dynamics, which can be attained by modeling at various levels of abstraction. Naturally, the chosen level should match the level at which neuronal recordings are made [1]. The activity patterns observed using voltage-sensitive dye (VSD) imaging reflect population activity at the mesoscopic (intra-areal) level [2], [3]. This suggests the application of mean-field models in which large numbers of neurons are averaged. Moreover, we are interested in the relation of neuronal dynamics to the spatial dimensions of the cortical sheet (and more generally to metric embeddings spanned by more abstract parameters, see [4], [5]). Neural field (NF) models [1], [6]–[11], in which the efficacy of synaptic coupling depends on the notion of distance between neurons or ensembles of neurons, are therefore our preferred choice. Here, we show that a minimalistic multiple-layer NF models can simulate mean VSD data in space and time with high accuracy. The model is an abstract functional description of VSD-recorded dynamics. Thus, it is in the first place phenomenological. However, its interpretation in biological terms allows to link its structure and parameters to the neuronal functional architecture.

The imaging data that we model showed: i) Two stationary stimuli (a square and an elongated bar) presented in rapid succession produce a pattern that signals propagation of activity across the bar's retinotopic representation in early visual cortex. ii) The obtained pattern was different from activity when the bar was flashed alone, and did not match the simple superposition of activities evoked by individually-presented square and bar stimuli. iii) Rather, we observed propagation of a wave front of activity that was also found when a square stimulus moved physically in visual space [12].

Based on the VSD imaging data [12], we hypothesized that a two-layer neural field [7]–[9] model can account for the findings i–iii. If so, this would imply that the feedback from higher brain areas is not a principal requirement to produce motion patterns across primary visual cortex upon presentation of a square and a bar flashed in rapid succession (as debated, e.g., in [12]–[17]).

Voltage sensitive dye imaging measures relative fluorescence changes that are linearly correlated to changes in membrane potentials [18],[19]). This technique currently allows recording of *in vivo* cortical activity at sub- as well as suprathreshold level with at least 10 ms temporal resolution and a spatial resolution of 50 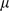
m across several millimeters of cortex. Hence, it is well suited to capture the real-time dynamics of millions of neurons at once. However, the signal does not distinguish between excitatory or inhibitory contributions to the overall activity. Therefore, our model explicitly assumes that the VSD signal reflects a mixture of activity from excitatory and inhibitory neuronal populations, and thus contrasts with studies that interpret VSD data as mainly reflecting excitatory activity (e.g., [20]–[22]). We expect to gain insights about the relative contributions of excitatory and inhibitory activities in the VSD signal, because the model allows separate inspection of its inhibitory and excitatory layers.

In the following, we first describe the underlying data and its preprocessing, our model structure, and our parameter identification procedure. Then we present the results including the model fit in comparison to further simplified models, the model prediction regarding similar yet novel stimuli, and the results of a standard linear stability analysis of the homogeneous solution of the model. We then follow with a discussion of the findings in relation to alternative modeling approaches, the physiological interpretation of our model, and the role of excitation and inhibition in the model. Finally, we consider our results in the context of hypotheses concerning the cortical representation of motion and the origin of the line-motion illusion.

## Methods

### Data

The data underlying this study were recorded by Jancke et al. at the Department of Neurobiology at the Weizmann Institute of Science in Israel using VSD imaging of cat visual cortex [12], [18], [23]. Animals were initially anesthetized with a mixture of ketamine (15 mg 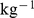) and xylazine (1mg 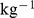). After tracheotomy, animals were respirated and anesthetized with 1.5% halothane (0.8% during recordings) in a 1∶1 mixture of 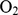 and 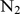
O. The animals were paralyzed with pancuronium bromide (0.2 mg 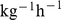, intravenously). Area 18 was stained for 2.5–3 h with the voltage-sensitive dye RH-1691. Its molecules bind to the external surface of excitable membranes and transform changes in membrane potential into changes in fluorescence intensity, which is correlated linearly with membrane potentials of layer 2 and 3 cortical neurons [19], [24]. Using a high-speed camera, the VSD signals were recorded with a temporal resolution of 9.6 ms. Stimuli were presented binocularly. The projection of the area centralis to the monitor screen was determined using a fundus camera. If necessary, projection of the eyes was converged using a prism in front of the eye that was ipsilateral to the recorded hemisphere. To control for possible eye drift during the experiment, the position of the area centralis and receptive field positions were measured repeatedly. The stationary stimuli were a square of 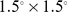 and a bar of 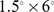. Both stimuli were aligned with their upper edges. The line-motion (LM) stimulus consisted of a square briefly presented before the bar. Additionally, squares were moved vertically with 4, 8, 16, and 32 deg/s. Stimuli were placed at 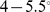 eccentricity. In the LM setting the square was presented for 50 ms followed by an inter-stimulus interval of 10 ms, after which the bar was presented for 130 ms. In single presentations the square and the bar were displayed with the same individual timings as in the LM condition.

The imaged signals (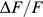) reflect relative changes in fluorescence compared to baseline: Imaging data were normalized by its DC level during pre-stimulus period (200 ms) for each pixel; heart-beat and respiration-related artifacts were removed by dividing by the average of “blank” signals recorded in absence of stimulation. For more details about the imaging methods we refer to [12].

**Figure 1:**
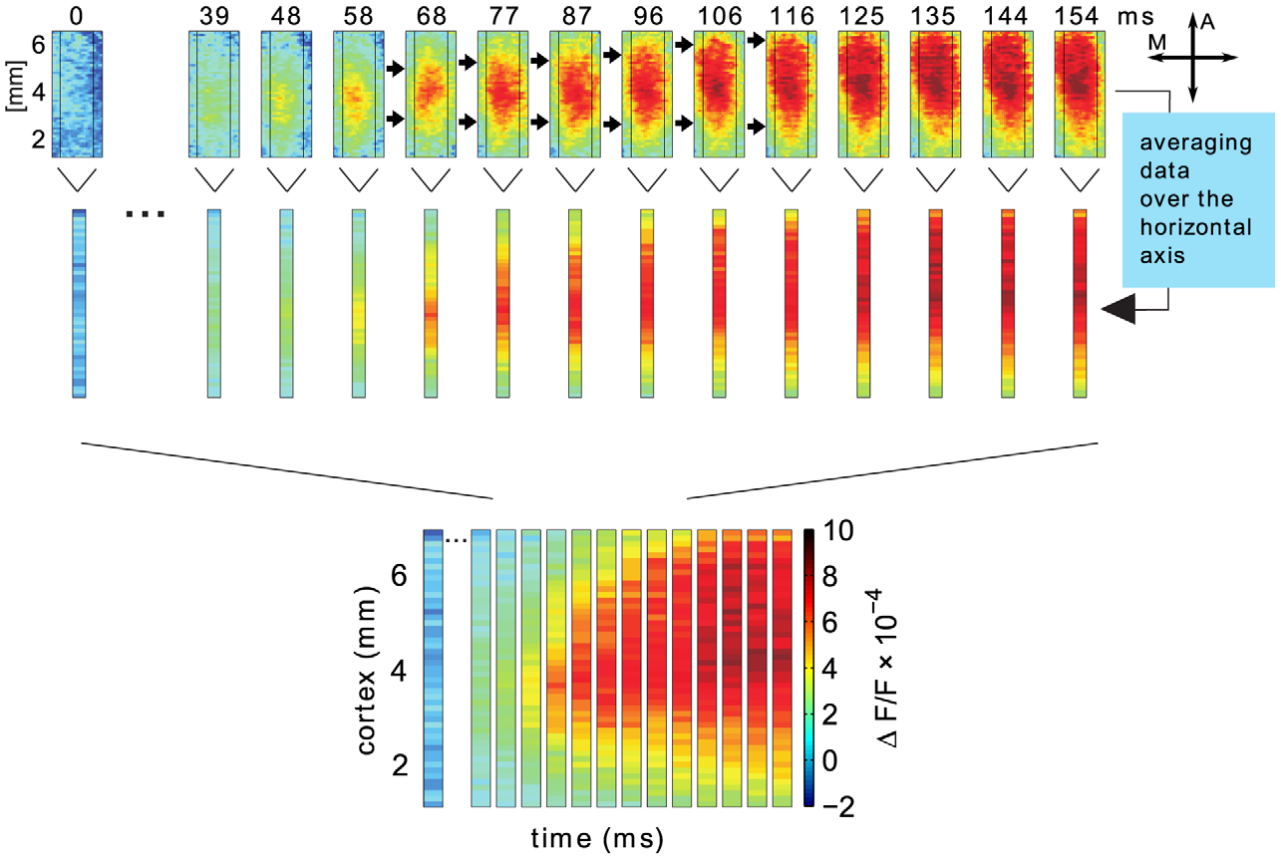
Deriving a space-time diagram of the VSD signals. The two-dimensional VSD image frames were averaged along the horizontal axis (thin vertical lines highlight the spatial region considered for averaging) to reduce the data to one spatial dimension. The vertical axis of the camera frame corresponds to the visual field azimuth representation in the cortex, where stimuli in the lower visual field are represented in anterior direction [27]. The exact position of the optical chamber was guided by the layout of cat area 18 retinotopic map around the trepanation site (Horsley-Clarke 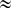
A0–A10, 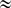
1–5 mm from midline) and verified through sampling of several receptive field locations prior to and partly during imaging sessions. The fact that the bar representation is drawn-out towards anterior (see upper black arrows), starting from the square representation (frames 48–58 ms) is interpreted as a motion signal. It also supports the correct settings as the square was presented at the upper end of the bar in visual field coordinates. In this study, we used the space-time activity patterns for modeling, with the spatial dimension on the ordinate and time on the abscissa. The colorbar indicates levels of activity (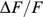, see “Methods”), A = anterior, M = medial.

We restrict our analysis to the first 250 ms of the VSD recordings during which propagation of activity was observed. The VSD data are sequences of frames that image 3 mm
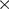
7 mm of cortex. This area was discretized into 24
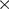
50 pixels (px). We denote the fluorescence change represented by a pixel at position 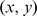 at time 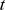 by 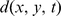. As the stimulus representations in this study vary only along the posterior-anterior cortical axis we reduced the dimensionality of the data by averaging 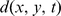 along the medial-lateral cortical axis (as done, e.g., in [25]), see Figure 1. For each vertical position 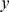 and time step 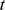, we define 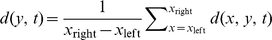, where 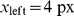 (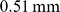) and 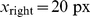 (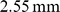). To eliminate the low signal-to-noise at the border of the representations, activity was averaged across the central pixels 4–20. These values were defined as mean 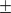 standard deviation of a Gaussian function fitted to the distribution of activity values across x-axis in the flashed bar condition (see Figure S1 in Text S1). However, the exact choice of these positions did not affect the results. Given this dimension reduction, the variable 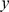 is from hereon referred to as 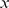 for clarity. Finally, the VSD data were additionally normalized using the mean level of activity when no stimulus was presented as reference. That is, for each stimulus condition the activity was averaged over the first 20 ms and all 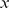 and the result was subtracted from the data.

### Model

We aimed for a model that quantitatively captures the spatio-temporal cortical dynamics observed by VSD imaging in response to the stimuli described above. The model should have as few parameters as possible, and these parameters should allow for functional interpretations (e.g., in terms of lateral interactions and time constants). The model should be at the same level of abstraction as the neuronal data. As our data reflect the dynamics of neuronal populations and describe the spread of activity across the cortical sheet, NF models are an appropriate choice [1], [4], [5], [21], [26].

The model is one-dimensional as the dynamics of interest evolve in one dimension along the (apparent) movement direction as described above. Our two-layer NF is governed by the following system of integro-differential equations:
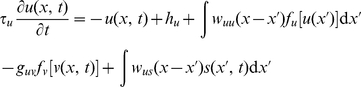
(1)

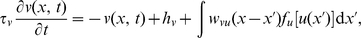
(2)where 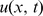 and 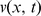 denote the mean membrane potentials of model neurons at cortical position 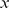 and time 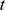 in the excitatory and inhibitory layer, respectively, and 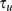 and 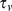 are the corresponding time constants. The resting potentials are determined by 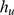 and 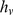. The transfer functions (or axonal response functions) 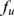 and 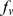 are represented by sigmoidal functions of the membrane potential, which relates depolarization to firing rate. For instance, 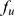 is given by
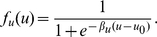
(3)The parameter 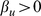 describes the slope of the response function and 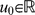 its threshold value. The transfer function 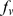 is parameterized analogously. The efficacy of the synaptic connectivity between two positions 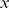 and 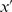 is assumed to be translation invariant and isotropic. It is given by gain factors 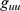, 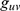, 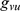 and synaptic strength (weight) functions 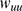 and 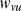 of Gaussian shape. For example, the coupling 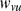 from the excitatory to the inhibitory layer is modeled by
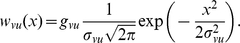
(4)The parameter 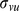 controls the width and 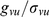 controls the strength of the interactions. Eccentricity in the imaged region of area 18 (see legend Figure 1) is approximately linear along the vertical meridian [27], [28] mapping one degree of the visual field to 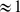 mm of cortex. The external visual stimulus mapped accordingly to cortical coordinates is denoted by 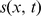, and the afferent input is computed by smoothing the stimulus by convolution with a Gaussian function 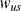. The afferent input couples only into the excitatory layer, and the inhibition is coupled into the excitatory layer locally.

There are several reasons for the local coupling from the inhibitory to the excitatory layer. From a technical point of view, we want our model to have as few parameters as possible. Equations 1 and 2 can be viewed as the minimal extension of the original Amari neural field model [9] to distinct dynamics for excitatory and inhibitory neurons (see also Eqn 10), allowing for different time constants and making it consistent with Dale's law (we added the response function 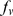 such that all communication between neurons can be interpreted as being encoded by mean firing rates). The resulting model is similar to the dynamics considered in [4], and it can be argued that the local coupling resembles physiology because the majority of interneurons projects locally [29].

The presented NF model is parameterized by 15 values. However, because all model neurons communicate only through the transfer function 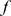 (see Eqns 1 and 2), shifting of resting potentials 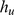 and 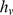 can be compensated by changing the transfer function thresholds 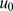 and 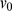 in Eqn 3 such that the dynamics are not affected. Thus the parameters 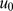 and 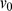 are redundant.

The VSD data reflect the activity of both excitatory and inhibitory populations of neurons. Accordingly, we define the modeled VSD signal 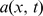 to be a combination of the field's excitatory and inhibitory activities. We assume an affine linear mixture
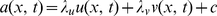
(5)with the non-negative coefficients 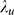 and 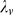 controlling how strongly excitatory and inhibitory activity is reflected in the dye signal, and 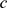 is some offset [30], [31]. We do not explicitly consider units of measurement to keep the notation uncluttered. Formally, the dye signal 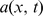 is measured in change of fluorescence intensity 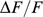 and the potentials in mV. Therefore, 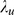 and 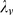 have units 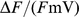 and 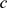 is measured in 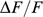.

The time delay between the stimulus presentation and the response onset in the VSD data is a sum of the time the neuronal signal needs to travel from the retina to the primary visual cortex and the time the neuronal populations in the primary visual cortex need to build up a detectable activity. A fixed retino-cortical time delay of two time frames (19.2 ms) was used in our model to align the response onsets of the model and the VSD data.

In our numerical experiments, we iterated the dynamical systems defined by Eqns 1 and 2 starting from the initial conditions 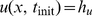 and 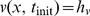 for all 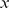. Then we let the system relax for some time period in the absence of afferent input. When we present our results, the time 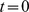 is after this relaxation phase. In our model, we had to discretize the spatial dimension. We simulated 150 spatial positions. The center 50 positions were mapped to the 50 pixels of the VSD image data. The other positions were added to avoid boundary effects and are not shown in the results.

### Model parameters identification procedure

In order to explore the system parameter space we adopted a grid search procedure on a reduced parameter set. The reduced set contained 10 parameters 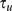, 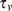, 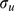, 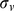, 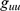, 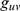, 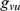, 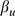, 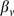 and 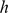, where the excitatory and inhibitory resting potentials were assumed to be equal (i.e., 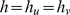). We defined a grid with 3 points (chosen by educated guess, see Table 1, third column) for each parameter, which results in total to 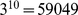 parameter configurations. The remaining parameters 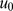, 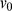, 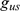 and 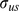 were fixed to physiologically plausible values. The transfer function thresholds 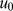 and 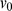 were both set to −40 mV (see discussion of parameter redundancy in the previous section). The feed-forward gain 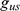 was set to 70 to yield maximal amplitude of the input signal of about 60 mV. According to the literature, one retinal position is represented in our region of interest by a population of cortical neurons distributed around the mean position with a standard deviation of approximately 0.6 mm [28]. The parameter 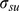, determining the feed-forward smoothing of the input (as parameter of 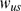, see Eqns 1 and 4), was therefore fixed to 4 pixels in the discretized model, which corresponds to a value of 0.51 mm.

**Table 1:** Summary of the model parameters.

Parameter	Description	Grid points	Value
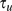	time constant for excitatory layer	9.6, 19.2, 28.8 ms	19.2 ms
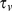	time constant for inhibitory layer	9.6, 19.2, 28.8 ms	28.8 ms
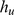	resting potential for excitatory layer	−100, −80, −60 mV	−60 mV
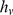	resting potential for inhibitory layer	was set equal to 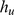	−60 mV
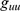	self-excitation gain	50, 125, 200	125
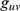	inhibition of excitatory layer	50, 125, 200	50
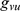	excitation of inhibitory layer	50, 125, 200	125
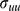	width of excitatory-excitatory kernel	0.64, 1.27, 1.91 mm	1.27 mm
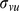	width of excitatory-inhibitory kernel	0.64, 1.27, 1.91 mm	1.27 mm
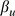	transfer function steepness for excitatory layer	0.05, 0.1, 0.15	0.15
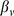	transfer function steepness for inhibitory layer	0.05, 0.1, 0.15	0.1
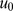	transfer function threshold for excitatory neurons	−40 mV	−40 mV
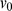	transfer function threshold for inhibitory neurons	−40 mV	−40 mV
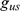	feed-forward gain	70	70
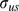	width of feed-forward smoothing kernel	0.51 mm	0.51 mm

The second column gives the description of the parameters. Values used for the grid search procedure are given in the third column. The selected parameter values are shown in the last column.

After we have simulated our NF model with a given set of model parameters and obtained the spatio-temporal patterns of the excitatory and inhibitory layer in response to the stimuli used for system identification, we can compute the values 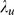, 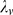, and 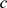, see Eqn 5, using the ordinary least-squares (OLS) method under the constraints 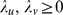. That is, we solve
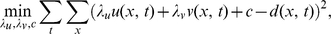
(6)where (with a slight abuse of notation) 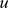, 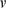, and 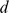 refer to the concatenated signals of all stimulus configurations considered in the optimization procedure. This yields the optimal values for 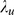, 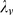, and 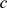 in terms of the mean squared error between aggregated model signal 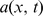 and observed dye patterns 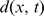 for the given spatio-temporal patterns of the excitatory 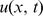 and inhibitory 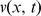 layer. From the solution, we get the aggregated signal
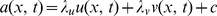
(7)and obtain the mixture coefficient
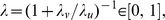
(8)of excitation and inhibition. The 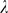 coefficient indicates that the simulated signal is comprised of 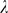 excitation and 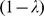 inhibition (under the assumption that the values of 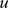 and 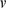 vary in the same interval). For optimization of the system, only four of the seven available stimulus configurations were used, namely the flashed square, the flashed bar, the LM stimulus, and a square moving at 32 deg/s. The other three stimulus conditions, squares moving at different speeds, were used to test whether the model generalizes to unseen, but related stimuli.

We selected parameter configurations from the sets produced by the grid search procedure according to the following criteria: (1) the system with given parameters is stable (see section “Analysis of Stability”); (2) after stimulus presentation, the simulated activity eventually decays to zero; (3) the correlation coefficient between the dye and the simulated data is larger than 0.8; (4) the rate of activity change during the onset period (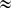
20–70 ms for the flashed square, LM and moving square stimuli; 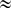
80–130 ms for the flashed bar stimulus) is similar in the modeled and measured responses.

In order to show that the model fit can be considerably improved by fine-tuning, we adjusted some model parameters using a randomized direct optimization algorithm. We used the selected parameter set from the grid search procedure (see Table 1) as starting point. We defined an objective function that quantifies the correlation coefficients between dye and simulated data as well as inhibition/excitation ratio:
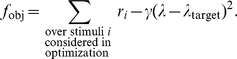
(9)The correlation coefficients 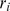 were computed between the dye and the simulated data for each stimulus configuration considered in the optimization and the trade-off parameter is set to 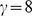.

This objective function is non-convex and multi-modal (i.e., there are undesired local optima). Our method of choice for such problems is the covariance matrix adaptation evolution strategy (CMA-ES) [32], [33]. The CMA-ES is an iterative, direct, stochastic optimization method and one of the most efficient biologically inspired search heuristics for real-valued optimization [34]. It has been successfully used to adapt neural field models [35]–[37] and is explained in detail in Text S1, section A.

## Results

### VSD imaging of cortical responses to the line-motion paradigm

The imaged visual cortical area included the complete retinotopic representation of the applied stimuli. Figure 1 shows the observed evolution of activity in response to the LM paradigm within single time frames as originally recorded (upper row). After the square was presented (frame zero), activity emerged and reached suprathreshold amplitudes around the thalamic input location (see region colored yellow/red in frames 48–58 ms; verified through spike recordings at various electrode positions [12]). After 60 ms the bar was presented, giving rise to activity that was gradually drawn-out (68–116 ms) along the retinotopic bar representation in the anterior cortical direction (see upper arrows and legend for more details). In contrast, the lower part of the responses showed no propagation (see lower row of arrows). The anteriorly propagating activity was interpreted as a motion signal that correlates to the perceived illusory line-motion as found in psychophysical studies [13]. Thus, instead of representing the bar at once, activity propagated across the retinotopic map. As shown in the original data, the same characteristics were obtained in multiple other experiments [12]. Since this effect occurred along the posterior-anterior cortical y-axis, we averaged each camera frame along the x-axis (see Figure 1) to enable a one-dimensional model approach (see 2nd row of Figure 1). Using these 1D frames, we depict activity in space-time diagrams that allow inspection of entire time courses (3rd row).

### Aggregated activity dynamics of both excitatory and inhibitory layers – emergence of propagating activity

**Figure 2:**
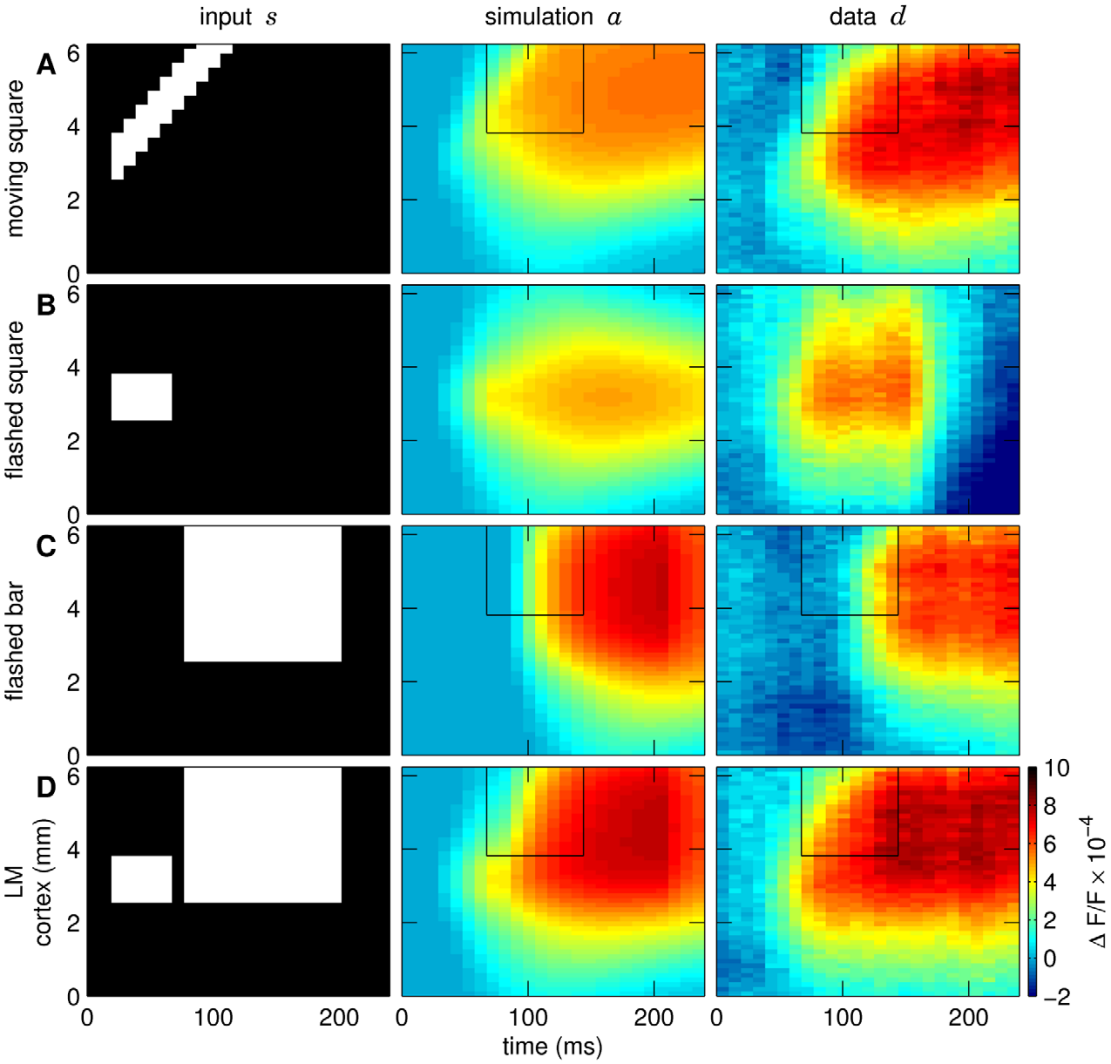
Visual inputs, modeled, and VSD responses. Visual stimuli mapped on cortical coordinates are shown in the first column: (A) moving square, (B) flashed square, (C) flashed bar, and (D) LM stimulus (square followed by bar). Second column: neural field responses to the respective stimuli for a mixing ratio 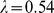 (see text) and the model parameters given in Table 1. Third column: corresponding dye signals. Vertical axes depict cortical coordinates, horizontal axes indicate time. Colors show response amplitudes as fractional changes in fluorescence. Rectangles in the space-time plots emphasize the region of interest. Here, a gradual propagation of high-level activity – from the lower left to the upper right – can be observed for both the model responses and the VSD data for real motion (A) and the LM condition (D) (see also Figure S2 in Text S1). The grid search procedure revealed one parameter configuration that satisfied the selection criteria (see “Methods”). The overall correlation coefficient between simulated and measured data was 0.85. The individual correlation coefficients computed between the simulated and measured responses to particular stimulus conditions were 0.83 for the square moving at 32 deg/s, 0.46 for the flashed square, 0.93 for the flashed bar and 0.93 for the LM stimulus condition.

The NF parameters found by grid search are summarized in Table 1. Figure 2 shows both the data and the NF responses. Activity in the model started with a delay of 19.2 ms (two time frames, see section “Model parameters identification procedure”), following low amplitude activity (light blue-green colors) that spread rapidly across several millimeters of cortex. With increasing amplitudes (yellow, red), the speed of the spread gradually decreased.

For the flashed square (Figure 2B), activity spread symmetrically around the stimulus input location. With either the flashed bar (Figure 2C) or LM stimulus (Figure 2D), nearly identical responses were found in the lower part (0–3 mm) of the space-time diagram, as there were no differences in stimulus inputs in this direction. In the upper part, however, as outlined by rectangles, there were crucial differences between these conditions around the elongated bar input location. In this region, we observed a gradual propagation of high-amplitude activity – from the lower left to the upper right for the LM stimulus but not for the single flashed bar (Figure 2C, a more detailed analysis is provided in Figure S2 in Text S1). Note that such propagation of high-amplitude activity was also observed both in the VSD and modeled responses to a moving square (Figure 2A). Thus, similarly to the LM stimulus, real moving input gave rise to a propagating wave front of cortical activity reporting physical motion across the retinotopic map (see Figure S2 in Text S1). In the model the LM effect results from the following mechanism: The activity in response to the flashed square is sustained after the stimulus offset and serves as a starting point for the response to the flashed bar. The bar-evoked excitation then propagates from this region producing a gradual spread of activity, drawn-out from the highest activity amplitudes. Hence, the LM effect persists in the model as long as the inter-stimulus interval is in the range of activity decay times after the square stimulus presentation.

**Figure 3:**
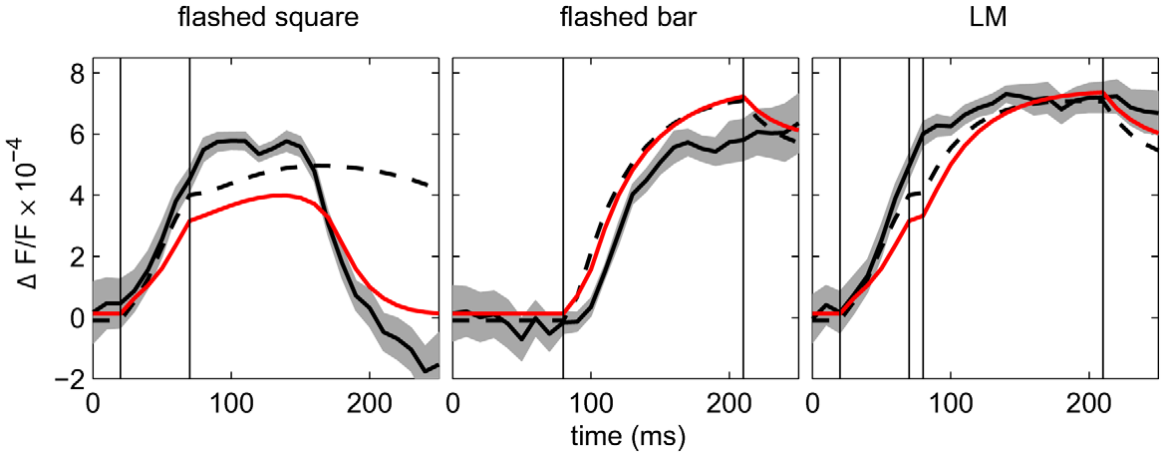
Time courses of aggregated activity of a single model neuron. Example traces were calculated around the input location of the single square (spatial coordinate 3 mm, see Figure 2). Solid black lines represent VSD data, shaded curves depict standard error, 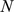 = 24 trials. Dashed lines represent modeled activity with parameters produced by grid search, red lines represent optimized modeled responses. The optimized parameter configuration can be found in Text S1, Table S1; see also Figures S3 and S4. Vertical lines indicate stimulus duration: square 20–70 ms, bar 80–210 ms.

Figure 3 shows the evolution of activity at the center of the cortex image (the model responses are given by dashed lines). Although the response to the square stimulus was sustained longer than the measured dye signal and the maximum amplitude was not fully reached in the NF, the model in general captured the time courses of the cortical responses to the different stimuli used. Moreover, a small adjustment of the parameters removed these discrepancies: Using evolutionary optimization (see “Methods” and Text S1, section A) the duration of the model response to the flashed square was greatly reduced (Figure 3, red lines), and the correlation coefficient for this single configuration increased to 0.81. The evolutionary optimization changed the parameters to 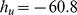 mV, 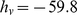 mV, 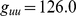, 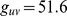, 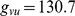, 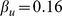, 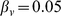 (see Text S1, Table S1).

### Nonlinear space-time interactions

**Figure 4:**
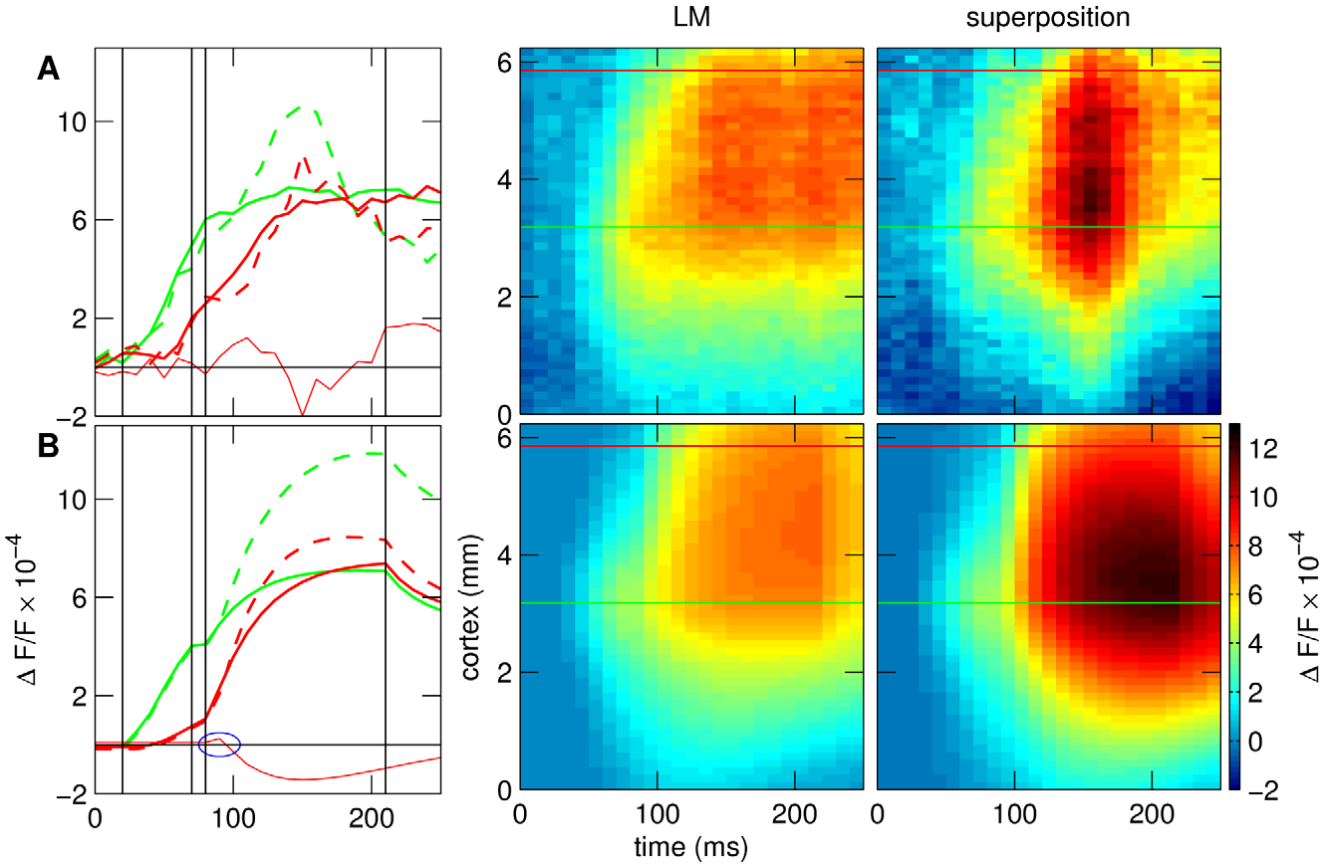
Comparison of the LM condition to the superposition of responses to the square and the bar alone. (A) VSD data, (B) Model. First column: time courses of activity at two single pixels at the center of the image (green line), and in the direction of activity propagation (red line). The superposition (dashed lines) differed from the corresponding response to the LM paradigm (solid lines) both for the recorded data and the model (see second and third column for entire space-time diagrams). In particular, the superposition showed a stronger response to the bar (from 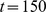 ms,31
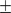
4% (mean 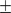
s.d.; n = 4) in the VSD data, 34
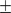
14.7% (n = 800 pixels) in the model). Responses to the bar were also delayed in the superposition, revealing a facilitatory effect in the beginning of the response (around 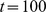 ms, VSD data: 24
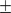
10% (n = 4), model: 5
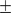
3.7% (n = 16, statistics computed with respect to the pixels that show this effect)). Note that the color code in this figure differs to the other figures as the superposition reached higher values than the single conditions.

We next analyzed whether nonlinear interactions are required to explain our findings to explain the space-time responses of the LM condition. The superposition of the responses to the single square and the bar alone differed from the response to their combined presentation in the LM stimulus (Figure 4). Note that the superposition also gave rise to propagation of activity as can be seen by the temporal offsets between the time courses at different locations (compare stippled red and green curves, Figure 4). However, there were marked deviations from linearity. The VSD LM response (Figure 4A) exhibited facilitation (around 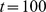 ms) and suppressive effects (around 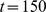 ms) as compared to the superposition of the single square and the bar alone. The significance of these effects was tested as follows. We defined three consecutive time intervals. The pre-stimulus interval, frames 1–4, the “facilitatory” interval, frames 5–12, and the “suppressive” interval, frames 13–20. First, we found no significant difference between all pixel values of the LM and the superposition in the pre-stimulus interval (Mann-Whitney U = 19808, 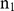 = 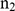 = 200, 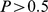, two-tailed). Second, we showed that the difference between the LM and the superposition is significant in the “facilitatory” interval (Mann-Whitney U = 64195, 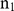 = 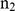 = 400, P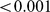, two-tailed) as well as in the “suppressive” interval (Mann-Whitney U = 64369, 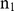 = 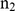 = 400, P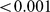, two-tailed). These effects are emphasized by thin red lines showing the difference of the LM response and the superposition (positive values indicate facilitation, negative values suppression). The model (Figure 4B) showed both of these effects, although the facilitatory effect (blue ellipse) was considerably smaller than in the measured data and was not observed at the center of the pattern (green line). We conclude that non-linear dynamics are necessary to explain the deviation from a simple superposition of the single square and bar representations.

### Comparison to reduced models

In order to verify that the different components of our model are necessary to reproduce the VSD-recorded dynamics, we tested several simplified models. We asked whether the spatio-temporal patterns could be simulated by a simple feed-forward model without lateral interactions, in which Gaussian smoothing of the input stimulus and low pass filtering produce the spread of activity (similar to the “G-waves” of Baloch and Grossberg [15]). It turned out that switching off lateral couplings and optimizing the parameters of the reduced model did not give satisfactory results.

Next, to investigate the role of inhibition and the necessity of different time constants for inhibition and excitation, we considered a single layer Amari-type NF [9],
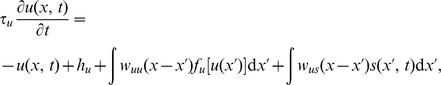
(10)where the kernel function 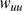 was either purely excitatory (Gaussian) or had a Mexican-hat shape. The latter models excitation and inhibition as the difference between two Gaussian kernels. Applying the grid search procedure to the reduced Mexican-hat model produced fairly good fits to the data with correlation values of up to 0.75 (compared to 0.85 for the full two-layer model), the individual correlation coefficients computed between the simulated and measured responses to particular stimulus conditions were: 0.55 for the square moving at 32 deg/s, 0.51 for the flashed square, 0.81 for the flashed bar, and 0.86 for the LM stimulus condition. The amplitudes of activity in response to smaller sized stimuli (i.e., flashed and moving squares) were too low. Testing this model on the moving stimuli produced responses that were too weak and too prolonged. The overall correlation coefficient between simulated and measured data for the four conditions with moving squares was 0.70. The individual correlation coefficients computed between the simulated and measured responses to particular stimulus conditions were 0.78 for the square moving at 4 deg/s, 0.84 for 8 deg/s, 0.69 for 16 deg/s, and 0.55 for 32 deg/s. As indicators for the goodness-of-fit, we computed the AIC (Akaike information criterion [38]) for the two-layer and the single layer NF. The AIC is given by 
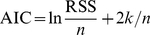
, where 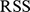 denotes the residual sum of squares, 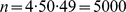 the number of data points (4 stimulus configurations, 50 spatial positions, and 25 time steps), and 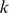 the number of parameters [38]. Note that the absolute values of the AIC cannot be interpreted. Although the two-layer model has more parameters, its AIC is smaller (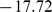 compared to 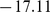) indicating a better fit. These results were obtained using the Mexican-hat kernel, the results for the simple Gaussian kernel were even worse.

In addition to the coarse grid search, we performed global optimization of the Amari-type field model using the CMA-ES (see Text S1, section A) without coming close to the fit quality of our two-layer model. Clearly, the fact that the system identification procedure did not find suitable parameters for a different model class does not prove that no suitable parameters for these alternative models exist (as such, non-existence proofs can be regarded as a general challenge in computational neuroscience). However, we regard the failure to fit the reduced models as a strong indication that lateral “intracortical” interactions and inhibition evolving with independent time constants are indeed necessary for the best fit of our data.

### Decomposing the model aggregated activity: Separate inspection of layers reveals their high correlation

**Figure 5:**
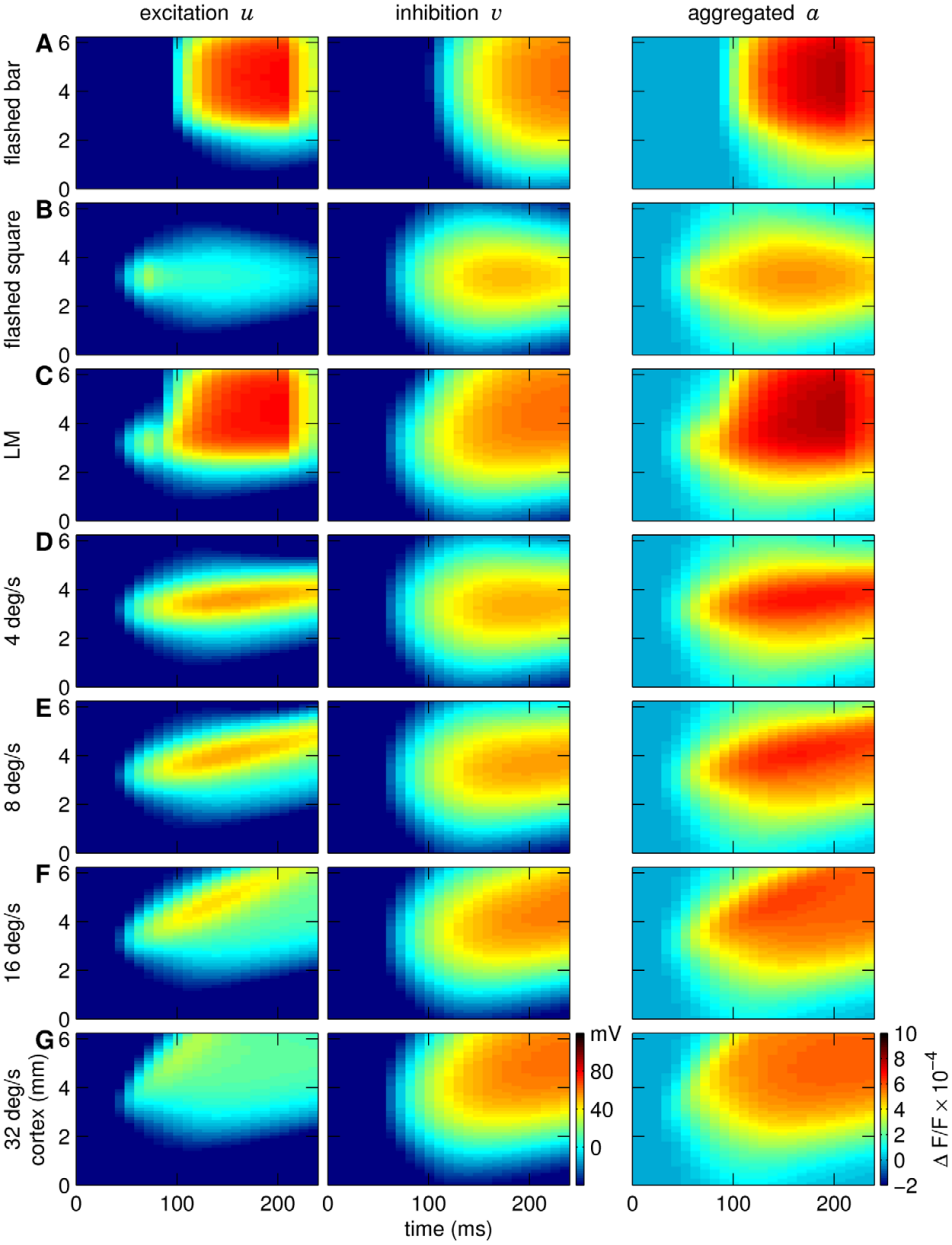
Decomposing NF activity into excitatory and inhibitory components. Columns from left to right: excitatory activity, inhibitory activity, aggregated signal (see Eqn 5). Stimulus conditions, rows A–G, as denoted on the left. Left colorbar applies to excitatory and inhibitory layers and indicates the modeled membrane potentials 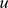 and 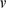, respectively. Right colorbar depicts response amplitudes as fractional changes in fluorescence intensity. The excitatory and inhibitory activity patterns were highly similar with correlation coefficients 0.79 (A), 0.81 (B), 0.81 (C), 0.84 (D), 0.84 (E), 0.86 (F), and 0.87 (G).

In Figure 5 the decomposed excitatory and inhibitory NF responses showed the observed gradual spread in both components.

The parameter 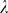, which reflects the mixing ratio of excitatory to inhibitory signals, was optimal for a value of 0.54 as revealed by our fitting procedure. This suggests that the aggregated signal was caused to a large extent by inhibitory processes. If we used for this parameter configuration either only 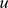 (or only 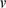) in the regression Eqn 6 (i.e., set 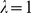 or 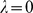, respectively), the quality of the fit significantly declined compared to the use of both layers' aggregated activities 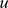 and 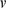 (verified by Wald-test [39], [40]. This indicated that elimination of the variable 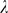 had a significant impact on the goodness-of-fit, 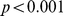). We computed the residuals 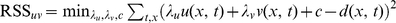 and 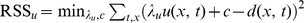, see section “Model identification procedure”. Let 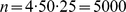 be the number of data points used for fitting the models (4 stimulus configurations, 50 spatial positions, and 25 time steps). We compared 
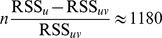
 to the 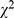 distribution [39], [40]. When interpreting this result one has to keep in mind that the residuals of our models are correlated because of the spatio-temporal structure of the data and their variances are not homogeneous.

**Figure 6:**
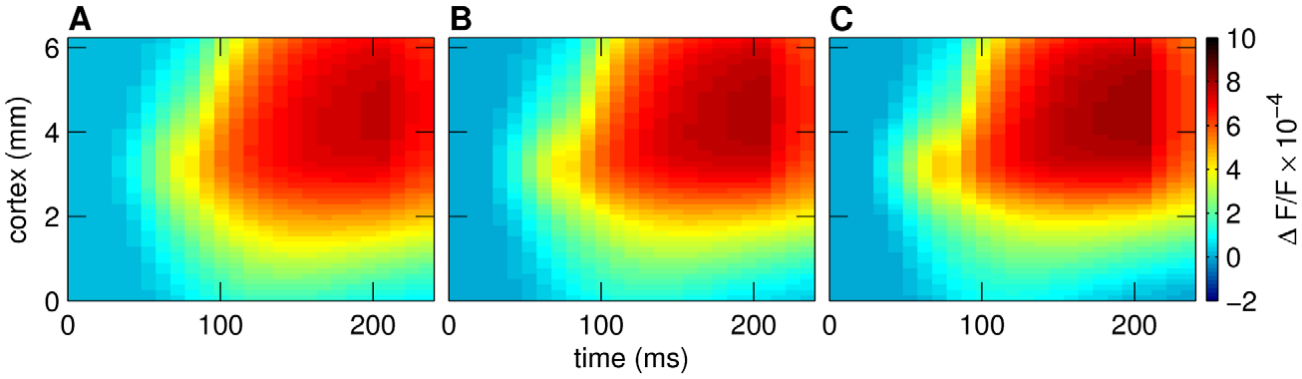
Different mixing ratios of excitatory and inhibitory activity. Aggregated model responses to the LM stimulus with (A) 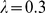, (B) 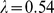, (C) 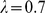. The value of 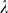 could vary over a wide range without considerably impairing the fit, i.e. decreasing the correlation coefficient. All mixing ratios of 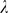 in the interval [0.3, 0.7] resulted in correlation coefficients larger than 0.8. The maximum 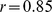 was obtained with 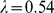 (B).

However, the choice of the mixing ratio 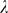 could vary over a wide range without a considerable decrease in fit quality as indicated by the high correlation coefficients for different mixing ratios (see Figure 6). This result was on the one hand surprising as we expected to reveal a critical value for this parameter. Consequently, we cannot estimate directly the relative fraction of excitatory and inhibitory signals in the VSD data. On the other hand, our results strongly suggest that both inhibitory and excitatory activity contribute significantly to the measured VSD responses as demonstrated by our statistical goodness-of-fit analysis. Moreover, our control experiments with simpler single layer models (see “Comparison to reduced models”) indicated that balanced interactions of the strongly coupled excitatory and inhibitory layer are required to produce the high correlation between the model aggregated spatio-temporal patterns and the VSD dynamics.

### Generalization to moving squares with different speeds

**Figure 7:**
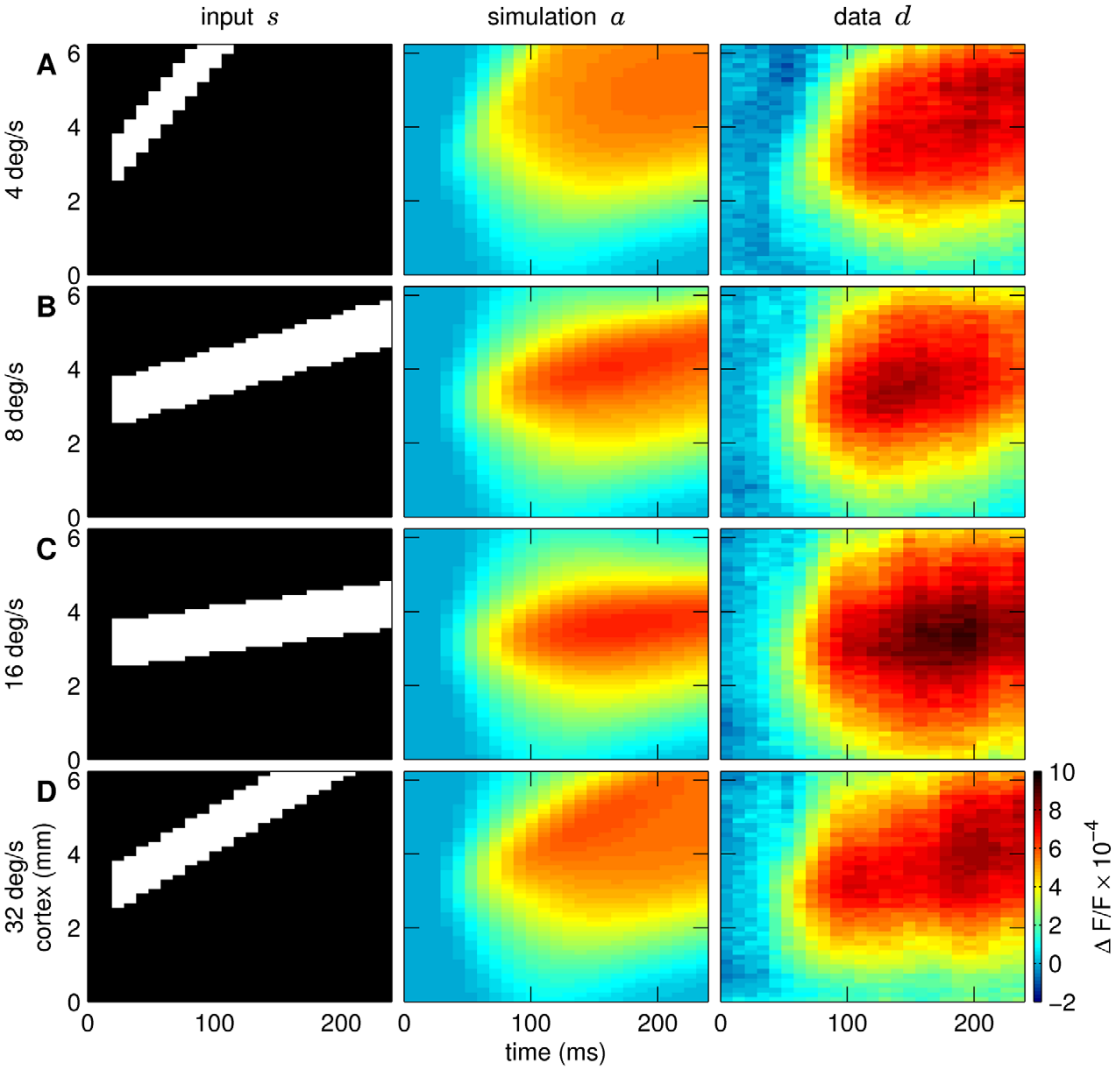
Model and VSD responses to moving squares with different speeds. Same conventions as in Figure 2. Stimulus speeds were: (A) 4, (B) 8, (C) 16, and (D) 32 deg/s. At lower levels of activity the spread of activity was uniform across all conditions (greenish colors). In contrast, at high-amplitudes (80% of maximal activation, red colors), the speed of propagation increased linearly with speeds: 0.004, 0.009, 0.02, 0.04 m/s. The individual correlation coefficients computed between the simulated and measured responses were 0.83, 0.92, 0.82, 0.83 for the square moving at 4, 8, 16, and 32 deg/s, respectively.

We finally tested the derived model using different stimuli that were similar to those used in the grid search procedure. Novel square stimuli moving at different speeds (4, 8, 16 deg/sec; the 32 deg/s stimulus was used for optimization) were fed into the model. The results, summarized in Figure 7, revealed that our model also reproduced these new spatio-temporal patterns. The correlation coefficient between simulated and measured data for the four moving square conditions was on average 0.79.

### Analysis of stability

For a better understanding of the dynamics of our model, we performed a standard linear stability analysis in the absence of afferent input (e.g., see [41]). We linearized the system governed by Eqns 1 and 2 near its homogeneous solution, which is described by 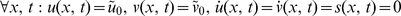 with scalars 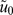 and 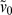.

The spatial dimension is considered in frequency space. The analysis yields two constraints that have to be met for the linearized system to be asymptotically stable:
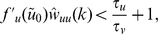
(11)

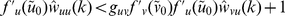
(12)for all spatial frequencies 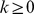, where 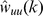 and 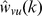 are the spatial Fourier transforms of the weight functions 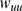 and 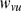 (e.g., 
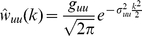
) and 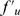 and 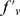 are the derivatives of the transfer functions. Substituting the parameters listed in Table 1 into the Inequalities 11 and 12 reveals that the homogeneous solution of our model is indeed asymptotically stable, which is a minimal requirement (for detailed derivation of Inequalities 11 and 12 see Text S1, section B).

## Discussion

This is the first time *in vivo* VSD patterns have been modeled quantitatively in space and time on a mesoscopic population level. Our proposed dynamical system is an abstract functional description of *in vivo* recorded VSD dynamics that correlate with changes in potentials across neuronal membranes and reflect the mass activity of a large pool of neurons.

Despite its rather few degrees of freedom, the NF model matches the VSD activity patterns evoked by briefly flashed visual stimuli including the LM paradigm. Our statistical analysis indicate that the two-layer structure of our model as well as the assumption that both excitatory and inhibitory activity contribute to the dye signal are necessary to obtain a fit with the highest correlation to the VSD imaging data.

The question arises whether our model is realistic in the sense that it could be implemented by the cortical network. We argue that the relevant model parameters are indeed within physiologically plausible ranges (see “Physiological interpretation of the model”). In this context, we suggest a significant contribution of inhibitory activity to the VSD dynamics.

Finally, the here presented modeling of V1 implies that feedback from higher brain areas is not necessary to produce activity patterns resembling the percept of illusory motion.

### Relation to alternative large-scale model

First, we compare our model to other in silico simulations that addressed the same experimental findings. Rangan et al. [20] simulated the VSD data from Jancke et al. [12] using a large-scale integrate-and-fire model, which consists of 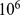 neurons modeling three types of channel conductances (NMDA, GABA, AMPA) and two types of connections (isotropic short-range and orientation-specific long-range connections) [42]. Importantly, both their model and ours simulate the LM effect without additional modeling of higher brain areas [20]. While our NF model was specifically designed to capture the LM data, the large-scale network developed by Rangan et al. [20] incorporated additional aspects of cortical processing on a different level of abstraction, as their large-scale model accounts for further experimental observations (for instance correlations between spontaneous activity and stimulus orientation [42], [43]) that cannot be addressed by our model, which is only as complex as needed to match our data. Therefore, it is not surprising that our model allowed a better fit per se.

Comparing the simulations in Rangan et al. [20] with our study, it becomes apparent that both approaches have a spatial and temporal integration problem: activity in response to the square stimulus is much less integrated over space and time than for the longer bar stimulus. The large-scale model [20] therefore included additional pre-processing in the lateral geniculate nucleus (LGN). Using such an LGN model that implements normalization, as proposed by several authors [25], [30], [44]–[47], may indeed help to enhance our fits but was not explicitly tested here.

In the large-scale model, the response following the bar arises about 20 ms earlier than the VSD-recorded responses. In contrast, the response to the square stimulus was 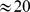 ms delayed. Using the constant retino-cortical time delay in our model, the simulated and measured signal onsets were aligned in all stimulus conditions. Our model thus has the advantage of capturing the timing of VSD signal onsets more accurately (see Figure 3).

### Possible extensions of our NF model

The model responses to both the flashed bar and LM condition fitted the observed VSD measurements. In contrast, for the single flashed and the moving squares the model revealed a discrepancy to the VSD data in the extent of lateral spread. One reason for this effect is our simple Gaussian smoothing that we used as a model for the retino-cortical processing. Increasing the kernel width 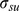 resulted indeed in a wider activity spread, but the tested widths were inappropriately large to match the common experimental findings. As another straightforward solution, we increased the widths of the coupling kernels 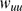 and 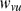, however, the grid search did not find models with an accurate fit for such wider kernels.

For the flashed square, prolonged activity was observed compared to the data. Importantly, tuning the gains 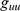, 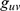, and 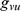, the resting potentials 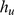 and 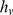, and the steepness of the transfer function 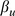 and 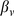 using evolutionary optimization eliminated the discrepancy (see Figure 3).

Finally, the flashed and moving square stimuli evoked model responses that were lower in amplitude than that measured. As stated in “Relation to alternative large-scale model”, using a normalization method [25], [30], [44]–[47] in the retino-thalamic processing step could be a suitable solution to adjust the amplitudes of the responses to the small- and large-sized stimuli. As we aimed at capturing essential nonlinear activity dynamics with the simplest form of our NF model, we also neglected further specific mechanisms of cortical processing like short-term synaptic plasticity and did not parameterize axonal propagation speed (see, e.g., [22], [31]). It should be noted that increasing the model complexity by introducing more parameters would certainly enhance the fit to the data. For instance, in a comparable version of the model, to account for spiking population data [48], we added a “shunting inhibition” term that allowed to produce a rapid spread of activity ahead of a moving square without pushing the model into the active mode [12]. However, the ability of the present model with its minimal complexity to fit and generalize suggests that the principles of two-layered architecture with lateral connectivity are sufficient to account for VSD-recorded dynamics.

### Physiological interpretation of the model

Our model is functional in the sense that it characterizes the dynamics of the VSD signal. Still, the model must also be plausible from a physiological point of view in the sense that it can be implemented by the underlying brain structures. In the following, we therefore discuss the ability of the cortical architecture to give rise to the model dynamics.

The NF model is a graded response mean-field approximation. As shown in the work of Eggert and van Hemmen [49], graded response models are able to describe the evolution of a population of spiking neurons in the case of slow dynamics. The VSD data used in this study lack these very fast ms-dynamics due to the frame duration and averaging and therefore, fulfills this requirement. The model accounts for the neuronal activity measured in cortical layers 2/3 and ignores other cortical structures. Furthermore, as it matches averages over several trials and numerous cell types with different connectivities and different neuronal response profiles, the neglected details affect the obtained model parameters. In this sense the model dynamics implicitly simulate all the factors that influence VSD-recorded dynamics.

The parameters 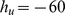 mV and 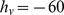 mV in the model can be associated with the mean resting membrane potentials of excitatory and inhibitory neurons, respectively. The physiological values reported in the literature span from 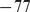 mV to 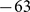 mV with a standard deviation of about 10 mV [50]–[54] for excitatory and inhibitory neurons in the cat primary visual cortex. Our modeled membrane potentials are in this range. However, it should be noted that since all model neurons communicate only through the transfer function 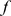 (see Eqns 1 and 2), shifting of resting potentials 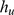 and 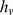 can be compensated by simply changing the transfer function thresholds 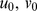 such that the system dynamics are not affected, see section “Model”. In the model, the mean membrane time constants 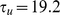 ms and 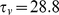 ms are in agreement with experimental data [51], [55].

The lateral connectivity is determined in the model by the Gaussian interaction kernels 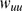 and 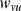 (see Eqn 4). As shown in Table 1, the values 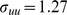 mm and 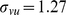 mm were used. The absolute values of the axonal extension of excitatory cells found in the cat primary visual cortex have been reported to reach up to 3.5 mm [56] or even up to 6–8 mm [57]. However, if our 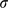-values are interpreted as measures of lateral extent of axonal-dendritic connections, they can be compared with the results of a recent quantitative study that measured and modeled the spatial and orientation preference distribution of labeled axonal bouton density of excitatory neurons in area 18 of the cat [58]. Modeling of the spatial distribution by a single Gaussian function revealed a 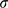-value of 0.6 mm, which is smaller than in our NF. However, their additional approach designed to differentiate between oriented and non-oriented components suggested values up to 1.1 mm for the non-oriented component, which is close to our results.

In contrast, inhibitory interneurons in the primary visual cortex act mostly locally [59], [60]. These interneurons are activated by widely spreading lateral excitatory connections (see [61] and [62], [63] for results for macaque and cat, respectively). This has been implemented in the NF by the local coupling of the inhibitory term into the excitatory field equation (see Eqns 1 and 2). Thus, although the coupling from the inhibitory to the excitatory layer is only local in our model, inhibition can act over a wide range.

### Excitation and inhibition in the VSD signal

We considered a mixing ratio of 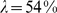, which stresses that the dye signal reflects both excitation and inhibition [30], [31]. This is in contrast to studies in which the signal is interpreted as caused by excitation only, but also to studies that presume only 25% inhibition [20]. The latter is based on the fact that about 25% of the neurons in the cat primary visual cortex are inhibitory (GABA-immunoreactive) [64]. We cannot exclude the possibility that the dye binds more strongly to inhibitory neurons, the number of active cells however, does in any case not necessarily reflect their functional impact. The NF dynamics are the result of processing across the closely coupled excitatory and inhibitory layers. We showed that the resulting dynamics were only achievable if both layers interact.

For example, as summarized in their review article, Ferster and Miller [65] pointed to the discrepancy between the observed contrast invariance of orientation tuning curves of simple cells and the notion of only weak cortical inhibition. In fact, recent intracellular in vivo recordings in cat have demonstrated strong inhibitory input [54] that may counteract cortical excitatory inputs in a push-pull manner [66].

### Relation to hypotheses of cortical representation of motion

Different amplitudes of the dye signal correspond to different levels of the degree of depolarization across the observed neural populations. Thus, with higher amplitudes, the probability rises that the signal reflects supra-threshold activity [12], [19]. Information about the stimulus trajectory should essentially be encoded at high amplitudes of activation, most likely as a propagating wave of spiking activity [12], [48]. In contrast, low amplitude activity may reflect initial passive spread [18] without a close coupling to the input speed of the stimulus. Therefore, we were particularly interested in the relationship between the speed of lateral propagation and the level of activity.

We found that the model did not reproduce cortical axonal conduction speeds of 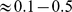 m/s [12], [67], [68]. Part of the problem to measure speeds of lateral spread is due to the fact that *in vivo* VSD imaging (as well as *in vivo* intracellular recordings) must measure weak deviations from baseline levels to capture the earliest input and are therefore confronted with low signal-to-noise-ratios. These initially very low activity levels observed in the VSD experiments (see [12] and also [68]) were indeed not high enough in the data to significantly contribute to our simple grid-based search algorithms if not explicitly parameterized. As we tried to keep our model as simple as possible we therefore did not further optimize these lowest activity levels. However, it is crucial to note that the measured propagation speeds are of postsynaptic origin. Thus, propagation of activity signals integrative properties of the neurons and the surrounding network, rather than reporting true axonal conduction speeds [25]. These network properties are reflected in the continuously rising VSD signal which we indeed captured in our NF model. Hence, in the model the speed of the initial low-amplitude activity spread (coded by the light blue color in Figure 7) demonstrates initially emerging activity within the network and shows only weak dependency on the stimulus speed as observed experimentally [12].

In conclusion, the model captures the main signatures of the spread of activity observed in the new data set including real motion. This generalization to novel stimuli similar to those used to determine the model parameters supports our choice of the neural field model architecture.

It remains an open question as to which neuronal mechanisms lead to the line-motion illusion. The dichoptic experiments of Hikosaka et al. [13] demonstrated that the retina and the LGN cannot be the processing stages where the LM effect arises. Instead, Hikosaka et al. argued that attentional effects are responsible for the line-motion sensation [13], [14]. Another proposal was that since area MT plays a major role in motion integration in humans and monkeys, the LM effect should arise along the dorsal pathway. For instance, Baloch and Grossberg [15] discussed a number of processing steps in their model of the V1–V2–MT–MST pathway that could give rise to the LM illusion.

A recent human fMRI study by Larsen et al. [16] demonstrated that MT+ activation in response to true motion was similar to activation following the presentation of a corresponding illusory moving stimulus. The three-stage theory proposed by these authors suggests that in the first stage higher areas solve the “correspondence problem” (i.e., identify two images as two successive views of the same object), while in the second stage MT+ computes the motion trajectory between these two object representations, and finally the computed trajectory is back-projected to V1 and filled in by a sequence of visual representations of the object [16]. Ahmed et al. [17] reported such feedback activation in area 17/18 from area 19/21 in ferrets using VSD imaging. The activation was motion-dependent and locked to the offset of the first stimulus in their apparent-motion paradigm. This feedback activated the path between subsequent retinotopic stimulus representations in area 17/18 and was interpreted as to play an important role in the computation of continuous motion.

In contrast, our model does not explicitly account for an impact of back projections from higher cortical areas. Nevertheless, these back propagating waves are slower than the initial LM effect characterized here by the immediate drawing-out of activity representing rapid motion [17]. However, we cannot exclude that particularly later parts of the VSD responses may involve feedback from higher visual areas.

The study by Jancke et al. [12] suggested that bottom-up processes are the main source of the initial line-motion activity. Our model confirms that lateral interactions in primary visual cortex are sufficient to generate responses to the illusory LM stimulus that are nearly indistinguishable from the responses to true motion.

## Supporting Information

Text S1 A) Covariance matrix adaptation evolution strategy; B) Linear stability analysis of the homogeneous solution.
(0.97 MB PDF)
