# Supplementary material for: A Dynamic Neural Field Model of Mesoscopic Cortical Activity Captured with Voltage-Sensitive Dye Imaging
Source: PLoS Comput Biol. 2010 Sep 9;6(9):e1000919. doi: 10.1371/journal.pcbi.1000919 (PMC2936513; doi:10.1371/journal.pcbi.1000919)
Supplement: Text S1 — A) Covariance matrix adaptation evolution strategy; B) Linear stability analysis of the homogeneous solution. (0.97 MB PDF) [file pcbi.1000919.s001.pdf]

Text S1

A Covariance matrix adaptation evolution strategy

Identifying suitable parameters of a neural field model is a non-linear optimization problem. In this study, we used a semi-automatic approach to solve it. We split the optimization problem into a linear and non-linear part:

1. After we have simulated our neural field model with some model parameters $\boldsymbol{x} \in \mathbb{R}^{13}$ and obtained the spatio-temporal patterns of the excitatory and inhibitory layer in response to the stimuli used for system identification, we can compute the values λ_u, λ_v, and c using the ordinary least-squares (OLS) method under the constraints $\lambda_u, \lambda_v \geq 0$, see Eq 6 in the main paper. This yields the optimal values for λ_u, λ_v, and c in terms of the mean squared error between aggregated model signal and observed dye patterns for the given the activities of the excitatory and inhibitory layer u and v, respectively.

2. For additional fine-tuning of the model parameters g_{uu}, g_{uv}, g_{vu}, β_u, β_v, h_u, and h_v we use a randomized direct optimization algorithm described below.

The objective function in the non-linear part of our model identification procedure is non-convex, non-differentiable, and multi-modal(i.e., there are undesired local optima). Our method of choice for such problems is the covariance matrix adaptation ES (CMA-ES) explained in the following.

The covariance matrix adaptation evolution strategy. Evolution strategies are randomized direct optimization algorithms [1–4]. They are one of the major branches of evolutionary algorithms, a class of algorithms drawing inspiration from principles of neo-Darwinian evolution theory.

Evolution strategies are iterative optimization methods. In every iteration (also referred to as *generation*), they sample a set of candidate solutions (the *offspring*) from a probability distribution over the search space, evaluate these points using the objective function f, and construct a new probability distribution over the search space. In typical ESs, this search distribution is parameterized by a set of candidate solutions, the *parent population*, and by parameters of the variation operators that are used to create the offspring from the parent population.

Evolution strategies are most frequently applied to real-valued optimization. Arguably the most elaborate ES for real-valued optimization is the covariance matrix adaptation ES (CMA-ES, [5–9]). It relies on on Gaussian mutations, that is, solutions are modified by adding random vectors drawn according to multi-variate zero-mean normal distributions. The CMA-ES is a variable metric algorithm adapting the shape and strength of its Gaussian search distribution. “[The] CMA-ESs represent the state-of-the-art in evolutionary optimization in real-valued $\mathbb{R}^n$ search spaces” [4]. This claim is backed up by many performance comparisons across different suites of benchmark problems [5,7,10–13].

The CMA-ES has been successfully applied for adapting neural dynamics in previous studies. In particular, the algorithm has been compared with the BFGS (Broyden -

Algorithm 1: rank-μ CMA-ES

initialize $\boldsymbol{m}^{(0)} = \boldsymbol{x}_{\text{init}}$, $\sigma^{(0)}$, evolution paths $\boldsymbol{p}_\sigma^{(0)} = \boldsymbol{p}_c^{(0)} = \boldsymbol{0}$ and covariance matrix $\boldsymbol{C}^{(0)} = \boldsymbol{I}$ (unity matrix)
// k counts number of generations
for $k = 0, \ldots$ **do**
    // create new offspring
    for $l = 1, \ldots, \lambda$ **do** $x_l^{(k+1)} \sim \mathrm{N}(\boldsymbol{m}^{(k)}, {\sigma^{(k)}}^2 \boldsymbol{C}^{(k)})$
    // evaluate new offspring
    for $l = 1, \ldots, \lambda$ **do** $y_l^{(k+1)} = f(\boldsymbol{x}_l^{(k+1)})$
    // recombination and selection
    $\boldsymbol{m}^{(k+1)} \leftarrow \sum_{i=1}^{\mu} w_i \boldsymbol{x}_{i:\lambda}^{(k+1)}$
    // step size control
    $\boldsymbol{p}_\sigma^{(k+1)} \leftarrow (1 - c_\sigma)\boldsymbol{p}_\sigma^{(k)} + \sqrt{c_\sigma(2 - c_\sigma)\mu_{\text{eff}}} {\boldsymbol{C}^{(k)}}^{-\frac{1}{2}} \frac{\boldsymbol{m}^{(k+1)} - \boldsymbol{m}^{(k)}}{\sigma^{(k)}}$
    $\sigma^{(k+1)} \leftarrow \sigma^{(k)} \exp\left(\frac{c_\sigma}{d_\sigma}\left(\frac{\|\boldsymbol{p}_\sigma^{(k+1)}\|}{\mathbb{E}[\|\mathcal{N}(\boldsymbol{0},\boldsymbol{I})\|]} - 1\right)\right)$
    // covariance matrix update
    $\boldsymbol{p}_c^{(k+1)} \leftarrow (1 - c_c)\boldsymbol{p}_c^{(k)} + \sqrt{c_c(2 - c_c)\mu_{\text{eff}}} \frac{\boldsymbol{m}^{(k+1)} - \boldsymbol{m}^{(k)}}{\sigma^{(k)}}$
    $\boldsymbol{C}^{(k+1)} \leftarrow (1 - c_{\text{cov}})\boldsymbol{C}^{(k)} + \frac{c_{\text{cov}}}{\mu_{\text{cov}}} \boldsymbol{p}_c^{(k+1)} {\boldsymbol{p}_c^{(k+1)}}^T + c_{\text{cov}}\left(1 - \frac{1}{\mu_{\text{cov}}}\right) \sum_{i=1}^{\mu} w_i \boldsymbol{z}_{i:\lambda}^{(k)} {\boldsymbol{z}_{i:\lambda}^{(k)}}^T$

Fletcher - Goldfarb - Shanno) method based on analytically derived gradients for learning the parameters of neural fields, and the CMA-ES clearly outperformed the quasi-Newton method [14, 15]. An application of the CMA-ES to modelling of human car driving behavior using neural attractor dynamics is presented in [16].

We can only expect very weak convergence results for optimization of non-differentiable and multi-modal objective functions in real-valued search spaces. Loosely speaking, if we store the best solution found so far in the optimization process, impose a lower bound on the step size[1], and assume that we just need to determine the optimal solution with an accuracy of $\epsilon > 0$, then the CMA-ES converges if the number of iterations goes to infinity, see [17] for details.

The CMA-ES in detail. For completeness, we briefly describe the CMA-ES as used in this article closely following [9]. For more detailed information, we refer to the literature [5–9].

In each generation k of the CMA-ES, which is shown in Algorithm 1, the lth offspring $\boldsymbol{x}_l^{(k+1)} \in \mathbb{R}^n$, $l \in \{1, \ldots, \lambda\}$, is generated by additive multi-variate *Gaussian mutation* and *weighted global intermediate recombination*:

$$\boldsymbol{x}_l^{(k+1)} \leftarrow \boldsymbol{m}^{(k)} + \sigma^{(k)} \boldsymbol{z}_l^{(k)}$$

with mutation $\sigma^{(k)} \boldsymbol{z}_l^{(k)} \sim \sigma^{(k)} \mathcal{N}(\boldsymbol{0}, \boldsymbol{C}^{(k)})$ and recombination $\boldsymbol{m}^{(k)} \leftarrow \sum_{l=1}^{\mu} w_l \boldsymbol{x}_{l:\lambda}^{(k)}$. Here $\boldsymbol{x}_{l:\lambda}^{(k)}$ denotes the lth best individual of the λ offspring ranked according to the values $y_l^{(k+1)} = f(\boldsymbol{x}_l^{(k+1)})$ of the objective functions $f : \mathbb{R}^n \to \mathbb{R}$. Considering the best μ of the

[1] To be more precise, in the CMA-ES we impose a lower bound on the global step size times the smallest eigenvalue of the covariance matrix of the mutation distribution.

offspring in the recombination implements non-elitist, rank-based selection. We use the standard choice for the recombination weights $w_l \propto \ln(\mu+1) - \ln(l)$, $\|\boldsymbol{w}\|_1 = 1, \boldsymbol{w} \in \mathbb{R}^\mu$.

The CMA-ES adapts both the $n \times n$-dimensional covariance matrix $\boldsymbol{C}^{(k)}$ of the normal mutation distribution as well as the *global step size* $\sigma^{(k)} \in \mathbb{R}^+$. The covariance matrix update has two parts, the rank-1 update considering the change of the population mean over time and the rank-μ update considering the successful variations in the last generation. The rank-1 update is based on a low-pass filtered *evolution path* $\boldsymbol{p}^{(k)}$ of successful (i.e., selected) steps

$$\boldsymbol{p}_c^{(k+1)} \leftarrow (1-c_c)\,\boldsymbol{p}_c^{(k)} + \sqrt{c_c(2-c_c)\mu_{\text{eff}}}\,\frac{\boldsymbol{m}^{(k+1)} - \boldsymbol{m}^{(k)}}{\sigma^{(k)}}$$

and aims at changing $\boldsymbol{C}^{(k)}$ to make steps in the promising direction $\boldsymbol{p}^{(k+1)}$ more likely by morphing the covariance towards $\left[\boldsymbol{p}_c^{(k+1)}\right]\left[\boldsymbol{p}_c^{(k+1)}\right]^T$. The backward time horizon of the cumulation process is approximately c_c^{-1}, where $c_c = 4/(n+4)$ is roughly inversely linear in the dimension of the path vector. The *variance effective selection mass* $\mu_{\text{eff}} = \left(\sum_{l=1}^{\mu} w_l^2\right)^{-1}$ is a normalization constant. The rank-μ update aims at making the single steps that were selected in the last iteration more likely by morphing $\boldsymbol{C}^{(k)}$ towards $\left[\boldsymbol{z}_{i:\lambda}^{(k)}\right]\left[\boldsymbol{z}_{i:\lambda}^{(k)}\right]^T$. Putting both updates together, we have

$$\boldsymbol{C}^{(k+1)} \leftarrow (1-c_{\text{cov}})\boldsymbol{C}^{(k)} + \frac{c_{\text{cov}}}{\mu_{\text{cov}}}\boldsymbol{p}_c^{(k+1)}\boldsymbol{p}_c^{(k+1)^T} + c_{\text{cov}}\left(1 - \frac{1}{\mu_{\text{cov}}}\right)\sum_{i=1}^{\mu} w_i \boldsymbol{z}_{i:\lambda}^{(k)}\boldsymbol{z}_{i:\lambda}^{(k)^T} \quad .$$

The constants c_{cov} and μ_{cov} are fixed learning rates. The learning rate of the covariance matrix update $c_{\text{cov}} = \frac{2}{(n+\sqrt{2})^2}$ is roughly inversely proportional to the degrees of freedom of the covariance matrix. The parameter μ_{cov} mediates between the rank-μ update ($\mu_{\text{cov}} \to \infty$) and the rank-one update ($\mu_{\text{cov}} = 1$). The default value is $\mu_{\text{cov}} = \mu_{\text{eff}}$.

An appropriate *adaptation of the mutation strength* (step size adaptation) is of utmost importance to balance exploration and exploitation during search and to reach an optimum with high accuracy [1, 2, 18]. The global step size $\sigma^{(k)}$ is adapted on a faster timescale. It is increased if the selected steps are larger and/or more correlated than expected and decreased if they are smaller and/or more anticorrelated than expected:

$$\sigma^{(k+1)} \leftarrow \sigma^{(k)} \exp\left(\frac{c_\sigma}{d_\sigma}\left(\frac{\|\boldsymbol{p}_\sigma^{(k+1)}\|}{\mathbb{E}[\|\mathcal{N}(\boldsymbol{0}, \boldsymbol{I})\|]} - 1\right)\right) \quad ,$$

where $\mathbb{E}[\|\mathcal{N}(\boldsymbol{0}, \boldsymbol{I})\|] = \hat{\chi}_n$ is the expected length of an n-dimensional random vector drawn from a zero mean Gaussian distribution with covariance matrix equal to the unit matrix and the (conjugate) evolution path is

$$\boldsymbol{p}_\sigma^{(k+1)} \leftarrow (1-c_\sigma)\,\boldsymbol{p}_\sigma^{(k)} + \sqrt{c_\sigma(2-c_\sigma)\mu_{\text{eff}}}\,\boldsymbol{C}^{(k)^{-\frac{1}{2}}}\frac{\boldsymbol{m}^{(k+1)} - \boldsymbol{m}^{(k)}}{\sigma^{(k)}}$$

with learning rate $c_\sigma = \frac{\mu_{\text{eff}}+2}{n+\mu_{\text{eff}}+3}$ and damping factor

$$d_\sigma = 1 + 2\max\left(0, \sqrt{\frac{\mu_{\text{eff}}-1}{n+1}}\right) + c_\sigma \quad .$$

The matrix $\boldsymbol{C}^{-\frac{1}{2}}$ is defined as $\boldsymbol{B}\boldsymbol{D}^{-1}\boldsymbol{B}^T$, where $\boldsymbol{B}\boldsymbol{D}^2\boldsymbol{B}^T$ is an eigendecomposition of $\boldsymbol{C}$ ($\boldsymbol{B}$ is an orthogonal matrix with the eigenvectors of $\boldsymbol{C}$ and $\boldsymbol{D}$ a diagonal matrix with the corresponding eigenvalues) and sampling $\mathcal{N}(\mathbf{0}, \boldsymbol{C})$ is done by sampling $\boldsymbol{B}\boldsymbol{D}\mathcal{N}(\mathbf{0}, \boldsymbol{I})$.

The CMA-ES is robust in the sense that it does not rely on tweaking of hyperparameters. The values of the learning rates and the damping factor are well considered and have been validated by experiments on many basic test functions [6]. *They need not be adjusted dependent on the problem and are therefore* no *hyperparameters of the algorithm.* Also the population sizes can be set to default values, which are $\lambda = \max(4 + \lfloor 3 \ln n \rfloor, 5)$ and $\mu = \lfloor \frac{\lambda}{2} \rfloor$ for offspring and parent population, respectively. If we fix $\boldsymbol{C}^{(0)} = \boldsymbol{I}$, the only hyperparameter to be chosen problem dependent is the initial global step size $\sigma^{(0)}$.

B Linear stability analysis of the homogeneous solution

We perform a standard linear stability analysis of the homogeneous solution of the DNF equations 1 and 2 of the main paper in the absence of afferent input. We follow the classic approach of von der Malsburg [19] (see also [20, 21]). The field equations without input

$$\begin{aligned} \tau_u \frac{\partial u(x,t)}{\partial t} &= -u(x,t) + h_u + \int w_{uu}(x-x') f_u[u(x',t)]dx' - g_{uv} f_v[v(x,t)] \\ \tau_v \frac{\partial v(x,t)}{\partial t} &= -v(x,t) + h_v + \int w_{vu}(x-x') f_u[u(x',t)]dx' \end{aligned} \tag{1}$$

are written as

$$\tau_u \dot{u}(x,t) = -u(x,t) + h_u + (f_u(u) * w_{uu})(x) - g_{uv} f_v(v) \tag{2}$$

$$\tau_v \dot{v}(x,t) = -v(x,t) + h_v + (f_u(u) * w_{vu})(x) \ . \tag{3}$$

Here $*$ denotes convolution and $\dot{u}(x,t) := \frac{\partial u(x,t)}{\partial t}$, $\dot{v}(x,t) := \frac{\partial v(x,t)}{\partial t}$, $(f_u(u))(x) := f_u(u(x,t))$, and $(f_v(v))(x) := f_v(v(x,t))$. We assume the weight functions to be translation invariant and isotropic.

We restrict our considerations to the homogeneous solution of the system with $u(x,t) = \tilde{u}_0$ and $v(x,t) = \tilde{v}_0$ for all x and t:

$$0 = -\tilde{u}_0 + h_u + f_u(\tilde{u}_0) W_{uu} - g_{uv} f_v(\tilde{v}_0) \tag{4}$$

$$0 = -\tilde{v}_0 + h_v + f_u(\tilde{u}_0) W_{vu} \ , \tag{5}$$

with $W_{uu} = \int w_{uu}(x')dx'$ and $W_{vu} = \int w_{vu}(x')dx'$. We consider small perturbations $\epsilon(x,t) = u(x,t) - \tilde{u}_0$ and $\eta(x,t) = v(x,t) - \tilde{v}_0$ at time step t:

$$\tau_u \dot{\epsilon}(x,t) = -(\tilde{u}_0 + \epsilon(x,t)) + h_u + (f_u(\tilde{u}_0 + \epsilon) * w_{uu})(x,t) - g_{uv} f_v(\tilde{v}_0 + \eta(x,t)) \tag{6}$$

$$\tau_v \dot{\eta}(x,t) = -(\tilde{v}_0 + \eta(x,t)) + h_v + (f_u(\tilde{u}_0 + \epsilon) * w_{vu})(x,t) \tag{7}$$

By replacing the transfer function f by its fist-order Taylor approximation the system is linearized:

$$\tau_u \dot{\epsilon} \approx -\tilde{u}_0 - \epsilon + h_u + (f_u(\tilde{u}_0) + \epsilon f_u'(\tilde{u}_0)) * w_{uu} - g_{uv}(f_v(\tilde{v}_0) + \eta f_v'(\tilde{v}_0)) \tag{8}$$

$$\tau_v \dot{\eta} \approx -\tilde{v}_0 - \eta + h_v + (f_u(\tilde{u}_0) + \epsilon f_u'(\tilde{u}_0)) * w_{vu} \ , \tag{9}$$

where the dependencies of x and t are removed for simplicity. We have

$$\tau_u \dot{\epsilon} \approx -\tilde{u}_0 - \epsilon + h_u + f_u(\tilde{u}_0) W_{uu} + f_u'(\tilde{u}_0)(\epsilon * w_{uu}) - g_{uv}(f_v(\tilde{v}_0) + \eta f_v'(\tilde{v}_0)) \tag{10}$$

$$\tau_v \dot{\eta} \approx -\tilde{v}_0 - \eta + h_v + f_u(\tilde{u}_0) W_{vu} + f_u'(\tilde{u}_0)(\epsilon * w_{vu}) \ . \tag{11}$$

By subtracting the homogeneous solution 4 and 5 from 10 and 11 we get

$$\tau_u \dot{\epsilon} \approx -\epsilon + f_u'(\tilde{u}_0)(\epsilon * w_{uu})(x,t) - g_{uv} \eta f_v'(\tilde{v}_0) \tag{12}$$

$$\tau_v \dot{\eta} \approx -\eta + f_u'(\tilde{u}_0)(\epsilon * w_{vu})(x,t) \ . \tag{13}$$

Now we apply the Fourier transform in the spatial dimension. Let $\hat{g}$ denote the Fourier transform of some function g. Eqns 12 and 13 become

$$\tau_u \dot{\hat{\epsilon}}(k,t) \approx -\hat{\epsilon}(k,t) + f_u'(\tilde{u}_0)\hat{\epsilon}(k,t)\hat{w}_{uu}(k) - g_{uv}\hat{\eta}(k,t)f_v'(\tilde{v}_0) \tag{14}$$

$$\tau_v \dot{\hat{\eta}}(k,t) \approx -\hat{\eta}(k,t) + f_u'(\tilde{u}_0)\hat{\epsilon}(k,t)\hat{w}_{vu}(k) \ , \tag{15}$$

where

$$\hat{w}_{uu}(k) = \frac{a_{uu}}{\sqrt{2\pi}} e^{-\sigma_{uu}^2 \frac{k^2}{2}}, \text{ for } w_{uu}(x) = \frac{a_{uu}}{\sqrt{2\pi}\sigma_{uu}} e^{-\frac{x^2}{2\sigma_{uu}^2}} \tag{16}$$

and $\hat{w}_{vu}$ is computed analogously. Writing Eqns 14 and 15 in vector form gives

$$\begin{pmatrix} \dot{\hat{\epsilon}}(k,t) \\ \dot{\hat{\eta}}(k,t) \end{pmatrix} \approx A(k) \begin{pmatrix} \hat{\epsilon}(k,t) \\ \hat{\eta}(k,t) \end{pmatrix}, \tag{17}$$

with

$$A(k) = \begin{pmatrix} -\tau_u^{-1} + \tau_u^{-1} f_u'(\tilde{u}_0)\hat{w}_{uu}(k) & -\tau_u^{-1} g_{uv} f_v'(\tilde{v}_0) \\ \tau_v^{-1} f_u'(\tilde{u}_0)\hat{w}_{vu}(k) & -\tau_v^{-1} \end{pmatrix}. \tag{18}$$

If the matrix $A(k)$ has two distinct eigenvalues, the homogeneous linear differential equation 18 can be solved as

$$\begin{pmatrix} \dot{\hat{\epsilon}}(k,t) \\ \dot{\hat{\eta}}(k,t) \end{pmatrix} \approx T(k) \begin{pmatrix} e^{t\lambda(k)_+} & 0 \\ 0 & e^{t\lambda(k)_-} \end{pmatrix} T^{-1}(k) \begin{pmatrix} \hat{\epsilon_0}(k) \\ \hat{\eta_0}(k) \end{pmatrix}, \tag{19}$$

with the initial values $(\hat{\epsilon_0}(k), \hat{\eta_0}(k))^T$.

The columns of the matrix $T(k)$ are the eigenvectors, where $\lambda(k)_+$ and $\lambda(k)_-$ are the corresponding eigenvalues. These can be compute by setting the characteristic polynomial $\lambda(k)^2 - \mathrm{tr}(A(k))\lambda(k) + \det(A(k))$ to zero:

$$\begin{aligned} 0 = \lambda(k)^2 - \lambda(k)\left(\tau_u^{-1} f_u'(\tilde{u}_0)\hat{w}_{uu}(k) - \tau_v^{-1} - \tau_u^{-1}\right) + \\ \tau_u^{-1}\tau_v^{-1}(1 - f_u'(\tilde{u}_0)\hat{w}_{uu}(k)) + \tau_u^{-1}\tau_v^{-1} g_{uv} f_v'(\tilde{v}_0) f_u'(\tilde{u}_0)\hat{w}_{vu}(k) \ . \end{aligned} \tag{20}$$

Solving this quadratic equation gives

$$\begin{aligned} \lambda_\pm(k) = \frac{1}{2}\left(\tau_u^{-1} f_u'(\tilde{u}_0)\hat{w}_{uu}(k) - \tau_v^{-1} - \tau_u^{-1}\right) \pm \Bigg[\frac{1}{4}\Big(\tau_u^{-1} f_u'(\tilde{u}_0)\hat{w}_{uu}(k) - \tau_v^{-1} - \tau_u^{-1}\Big)^2 \\ - \tau_u^{-1}\tau_v^{-1}\big(1 - f_u'(\tilde{u}_0)\hat{w}_{uu}(k)\big) - \tau_u^{-1}\tau_v^{-1} g_{uv} f_v'(\tilde{v}_0) f_u'(\tilde{u}_0)\hat{w}_{vu}(k)\Bigg]^{\frac{1}{2}} \ . \end{aligned} \tag{21}$$

For convenience, we write

$$\lambda_\pm = B \pm \sqrt{B^2 + C}. \tag{22}$$

If the real part of the largest eigenvalue is negative, then the system is asymptotically stable. In our system, we have to consider a Hopf bifurcation, in which a pair of complex conjugate eigenvalues ($\lambda_\pm$) of the linearization crosses the imaginary axis of the complex plane, and a saddle point bifurcation, where two fixed points, one stable and one unstable, collide and annihilate each other.

For the first case, we assume that the imaginary part of the eigenvalues is non zero. This means $B^2 + C < 0$, which implies $C < 0$. The bifurcation occurs when the real part of λ is zero (i.e., $\lambda_\pm = 0 \pm i\omega$, with $\omega \in \mathbb{R}, \omega \neq 0$). In this case, we have

$$\tau_u^{-1} f_u'(\tilde{u}_0)\hat{w}_{uu}(k) - \tau_v^{-1} - \tau_u^{-1} = 0 \tag{23}$$

and therefore

$$f_u'(\tilde{u}_0)\hat{w}_{uu}(k) = \frac{\tau_u}{\tau_v} + 1 \tag{24}$$

and applying this to the Gaussian kernel gives

$$f_u'(\tilde{u}_0)\frac{a_{uu}}{\sqrt{2\pi}} e^{-\sigma^2 \frac{k^2}{2}} = \frac{\tau_u}{\tau_v} + 1. \tag{25}$$

Thus, under the assumption that the discriminant $B^2 + C$ is negative, the system is asymptotically stable if $B < 0$ (the real part of the largest eigenvalue has to be negative, see above). That is, the following inequality has to be true

$$f_u'(\tilde{u}_0)\frac{a_{uu}}{\sqrt{2\pi}} e^{-\sigma^2 \frac{k^2}{2}} < \frac{\tau_u}{\tau_v} + 1. \tag{26}$$

Now we look at the second case where $B^2 + C \geq 0$. Then the system is obviously not asymptotically stable for $B \geq 0$, therefore we consider $B < 0$. The bifurcation point is $\lambda_\pm = \lambda = 0 + 0i$. From Eqn 20 we see that this equality holds if $C = 0$:

$$-\tau_u^{-1}\tau_v^{-1}\big(1 - f_u'(\tilde{u}_0)\hat{w}_{uu}(k)\big) - \tau_u^{-1}\tau_v^{-1} g_{uv} f_v'(\tilde{v}_0) f_u'(\tilde{u}_0)\hat{w}_{vu}(k) = 0 \tag{27}$$

For non negative discriminant and $B < 0$ this leads to the stability condition

$$f_u'(\tilde{u}_0)\hat{w}_{uu}(k) - 1 < g_{uv} f_v'(\tilde{v}_0) f_u'(\tilde{u}_0)\hat{w}_{vu}(k). \tag{28}$$

To summarize, the analysis reveals that the system is asymptotically stable if both, inequality 26 and inequality 28, are fulfilled.

References

1. Rechenberg I (1973) Evolutionsstrategie: Optimierung Technischer Systeme nach Prinzipien der Biologischen Evolution. Frommann-Holzboog.
2. Schwefel HP (1995) Evolution and Optimum Seeking. Sixth-Generation Computer Technology Series. John Wiley & Sons.
3. Beyer HG, Schwefel H (2002) Evolution strategies–A comprehensive introduction. Natural Computing 1: 3–52.
4. Beyer HG (2007) Evolution strategies. Scholarpedia 2: 1965.
5. Hansen N, Ostermeier A (2001) Completely derandomized self-adaptation in evolution strategies. Evolutionary Computation 9: 159–195.
6. Hansen N, Müller SD, Koumoutsakos P (2003) Reducing the time complexity of the derandomized evolution strategy with covariance matrix adaptation (CMA-ES). Evolutionary Computation 11: 1–18.
7. Kern S, Müller S, Hansen N, Büche D, Ocenasek J, et al. (2004) Learning probability distributions in continuous evolutionary algorithms – A comparative review. Natural Computing 3: 77–112.
8. Hansen N (2006) The CMA evolution strategy: A comparing review. In: Towards a new evolutionary computation. Advances on estimation of distribution algorithms, Springer-Verlag. pp. 75–102.
9. Suttorp T, Hansen N, Igel C (2009) Efficient covariance matrix update for variable metric evolution strategies. Machine Learning 75: 167–197.
10. Hansen N, Kern S (2004) Evaluating the CMA evolution strategy on multimodal test functions. In: Yao X, Burke E, Lozano JA, Smith J, Merelo-Guervós JJ, et al., editors, Parallel Problem Solving from Nature (PPSN VIII). Springer-Verlag, volume 3242 of *LNCS*, pp. 282–291.
11. Auger A, Hansen N (2005) Performance evaluation of an advanced local search evolutionary algorithm. In: Proceedings of the IEEE Congress on Evolutionary Computation (CEC 2005). IEEE Press, pp. 1777–1784.
12. Auger A, Hansen N (2005) A restart CMA evolution strategy with increasing population size. In: Proceedings of the IEEE Congress on Evolutionary Computation (CEC 2005). IEEE Press, pp. 1769–1776.
13. Whitley D, Lunacek M, Sokolov A (2006) Comparing the niches of CMA-ES, CHC and Pattern Search using diverse benchmarks. In: Parallel Problem Solving from Nature (PPSN IX). Springer-Verlag, volume 4193 of *LNCS*, pp. 988–997.
14. Igel C, Erlhagen W, Jancke D (2001) Optimization of dynamic neural fields. Neurocomputing 36: 225–233.

15. Schneider S, Igel C, Klaes C, Dinse H, Wiemer J (2004) Evolutionary adaptation of nonlinear dynamical systems in computational neuroscience. Journal of Genetic Programming and Evolvable Machines 5: 215–227.

16. Pellecchia A, Igel C, Edelbrunner J, Schöner G (2005) Making driver modeling attractive. IEEE Intelligent Systems 20: 8–12.

17. Rudolph G (1997) Convergence Properties of Evolutionary Algorithms. Hamburg: Kovač.

18. Schumer M, Steiglitz K (1968) Adaptive step size random search. IEEE Transactions on Automatic Control 13: 270–276.

19. von der Malsburg C, Cowan JD (1981) Theory of ontogenesis of orientation domains – Intracortical dynamics part. Technical Report 81–1, Department of Neurobiology, Max-Planck-Institut for Biological Chemistry, Göttingen.

20. Igel C (2003) Beiträge zum Entwurf neuronaler Systeme. Berichte aus der Informatik. Shaker Verlag.

21. Wennekers T (2002) Dynamic approximation of spatiotemporal receptive fields in nonlinear neural field models. Neural Computation 14: 1801–25.

Figure Legends

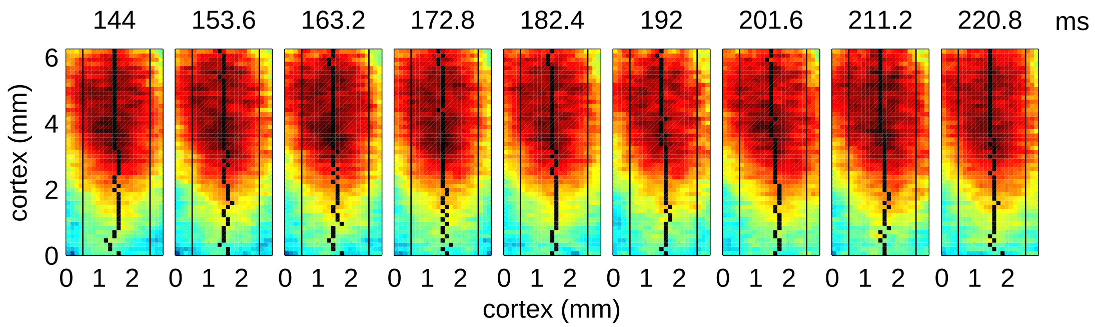

Figure S1. Activity centers of mass in response to the bar in the LM condition. Activity centers of mass computed for each horizonal row along y-axis are shown by black dots. The distribution of the centers of mass x-coordinates indicates no tilt in the representation of the stimulus along cortical posterior-anterior, 0 – 6 mm, y-axis. Thin vertical lines outline the spatial region of the standard deviation (mean across y-axis, 0.51 – 2.55 mm) used for averaging data across x-axis.

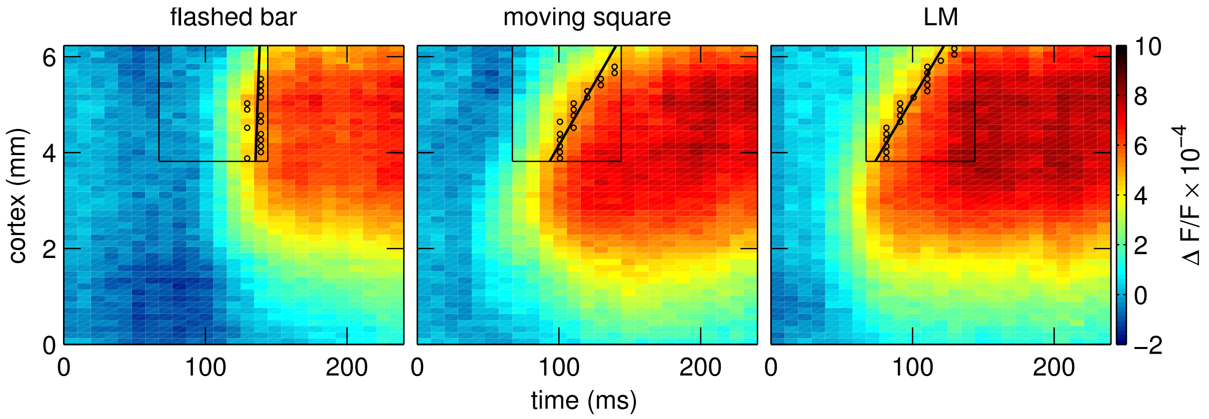

Figure S2. Trajectories of high-amplitude activity. VSD responses to flashed bar, moving square, and LM stimulus (square followed by bar). Rectangles in the space-time plots emphasize the regions of interest. The small circles denote the pixel positions where activity exceeded two standard deviations computed for all conditions shown here within the region of interest. Solid black lines visualize a linear regression computed using these positions. The regression lines show the differences in the trajectories. The hypothesis that the regression coefficients are zero, which indicates no propagation, is rejected for the moving square and the LM stimulus ($p < 0.01$), but cannot be rejected for the flashed bar.

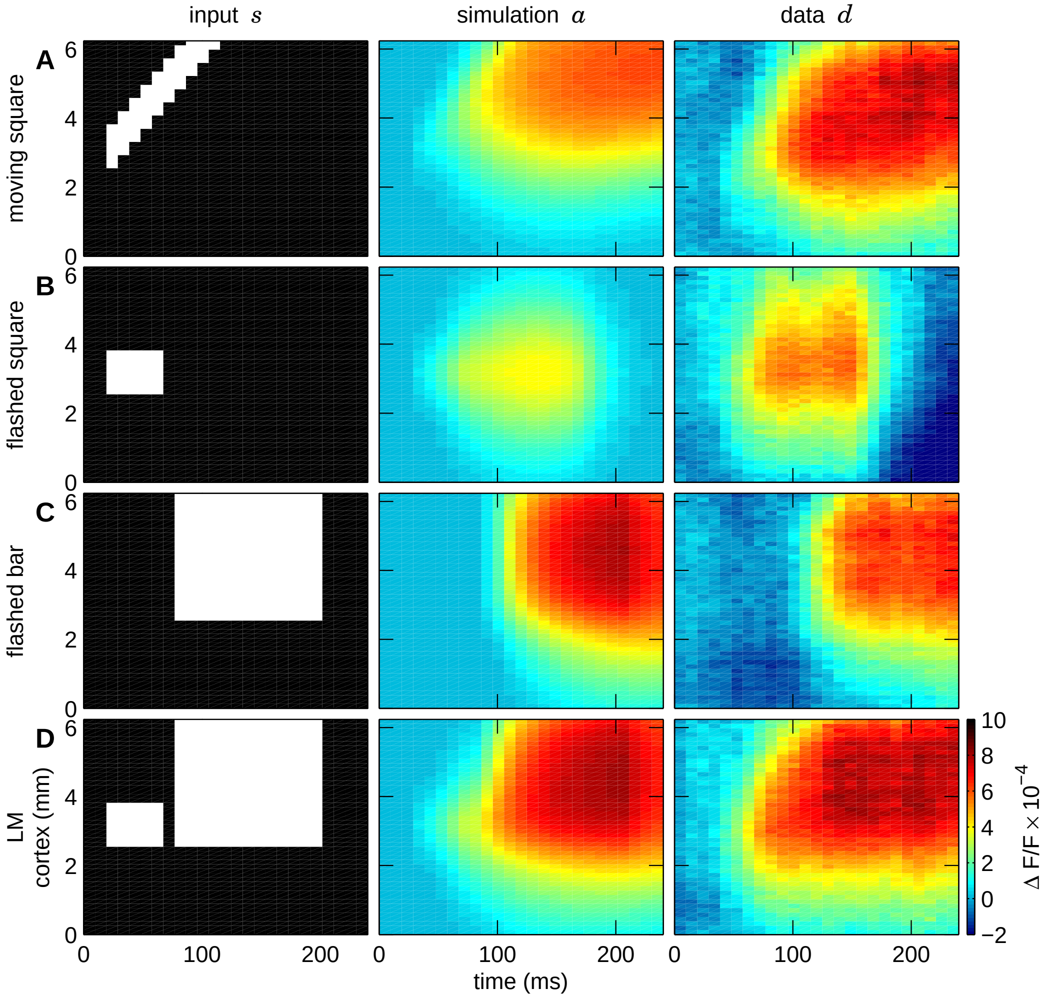

Figure S3. Visual inputs, responses of the optimized model and real VSD responses. Visual stimuli mapped on the cortical scale are shown in the first column: moving square (A), flashed square (B), flashed bar (C), and (D) LM stimulus. In the second column, the neural field responses of the optimized model (see parameter configuration in Table 1) to the respective stimuli are shown for a mixing ratio $\lambda = 0.50$. Comparison with Figure 1 of main paper reveals significant improvement of model fit to the flashed square stimulus (row B). The correlation coefficient between simulated and measured data for the four conditions with moving squares was 0.86. Individual correlation coefficients computed between the simulated and measured responses to particular stimulus conditions were 0.77 for the square moving at 32 (A) deg/s, 0.85 for the flashed square (B), 0.94 for the flashed bar (C) and 0.85 for the LM stimulus condition (D).

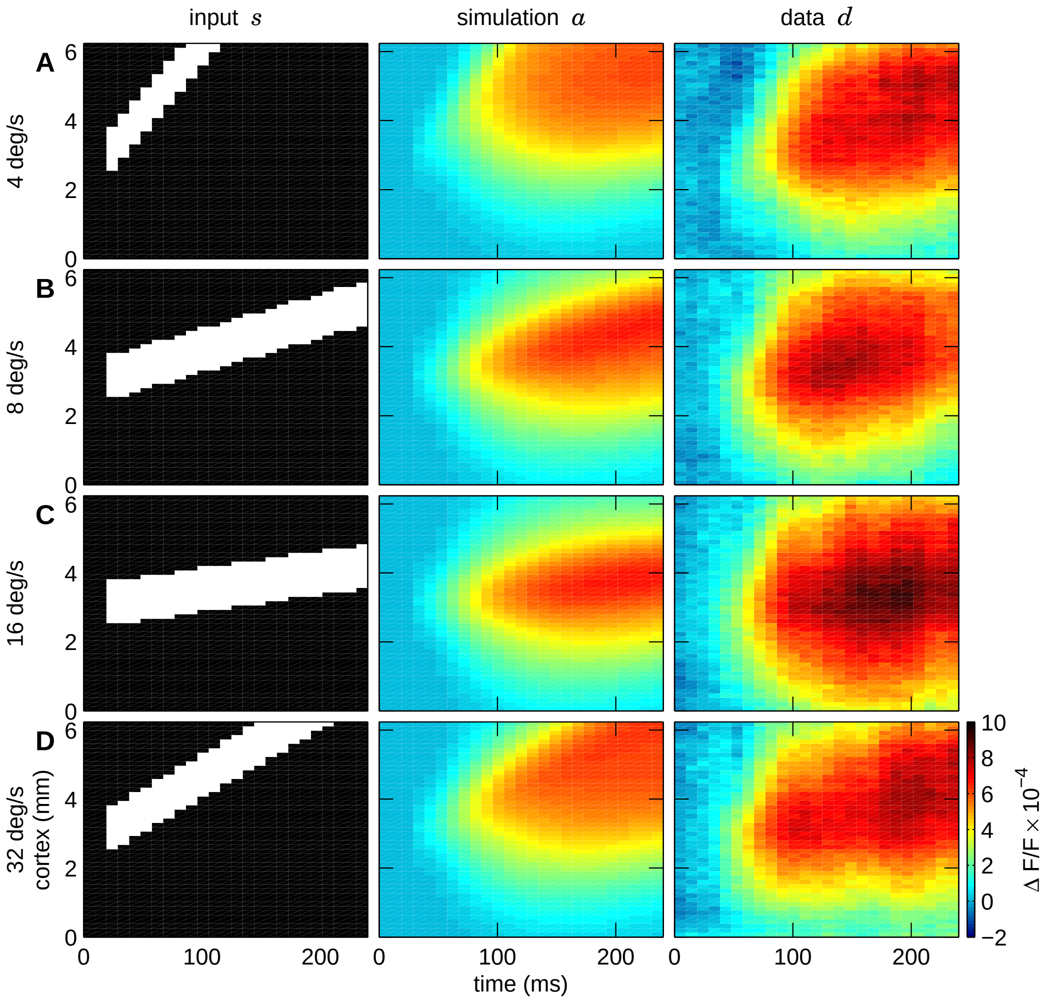

Figure S4. VSD and optimized model responses to moving square stimuli. Visual stimuli mapped on the cortical scale are shown in the first column, namely a moving square with 4 deg/s (A), 8 deg/s (B), 16 deg/s (C), and 32 deg/s (D), respectively. In the second column, the neural field responses to the respective stimuli are shown. The third column represents the corresponding dye signals obtained in the physiological experiments. The correlation coefficient between simulated and measured data for the four conditions with moving squares was 0.81, the individual correlation coefficients computed between the simulated and measured responses to particular stimulus conditions were 0.86 for the square moving at 4 deg/s, 0.89 for the 8 deg/s, 0.83 for the 16 deg/s, and 0.77 for the 32 deg/s.

Tables

Table S1. Summary of the optimized model parameters. The second column gives the description of the parameters. The third column tells whether the parameter was optimized. The parameter values are shown in the last column.

Parameter	Description	Optimized	Value
τ_u	time constant for excitatory layer	no	19.2 ms
τ_v	time constant for inhibitory layer	no	28.8 ms
h_u	resting potential for excitatory layer	yes	-60.8 mV
h_v	resting potential for inhibitory layer	yes	-59.8 mV
g_{uu}	self-excitation gain	yes	126.0
g_{uv}	inhibition of excitatory layer	yes	51.5
g_{vu}	excitation of inhibitory layer	yes	130.7
σ_{uu}	width of excitatory-excitatory kernel	no	1.27 mm
σ_{vu}	width of excitatory-inhibitory kernel	no	1.27 mm
β_u	transfer function steepness for excitatory layer	yes	0.16
β_v	transfer function steepness for inhibitory layer	yes	0.05
u_0	transfer function threshold for excitatory neurons	no	-40 mV
v_0	transfer function threshold for inhibitory neurons	no	-40 mV
g_{us}	feed-forward gain	no	70
σ_{us}	width of feed-forward smoothing kernel	no	0.51 mm
